# Engineered *Anopheles* Immunity to *Plasmodium* Infection

**DOI:** 10.1371/journal.ppat.1002458

**Published:** 2011-12-22

**Authors:** Yuemei Dong, Suchismita Das, Chris Cirimotich, Jayme A. Souza-Neto, Kyle J. McLean, George Dimopoulos

**Affiliations:** W. Harry Feinstone Department of Molecular Microbiology and Immunology, Bloomberg School of Public Health, Johns Hopkins University, Baltimore, Maryland, United States of America; Institut Pasteur, France

## Abstract

A causative agent of human malaria, *Plasmodium falciparum,* is transmitted by *Anopheles* mosquitoes. The malaria parasite is under intensive attack from the mosquito's innate immune system during its sporogonic development. We have used genetic engineering to create immune-enhanced *Anopheles stephensi* mosquitoes through blood meal-inducible expression of a transgene encoding the IMD pathway-controlled NF-kB Rel2 transcription factor in the midgut and fat-body tissue. Transgenic mosquitoes showed greater resistance to *Plasmodium* and microbial infection as a result of timely concerted tissue-specific immune attacks involving multiple effectors. The relatively weak impact of this genetic modification on mosquito fitness under laboratory conditions encourages further investigation of this approach for malaria control.

## Author Summary

Malaria is caused by the *Plasmodium* parasites which are transmitted by the *Anopheles* mosquitoes, and the mosquito's innate immune system plays an important role in blocking the parasite at several sporogonic stages. Our previous studies have shown that one of the major mosquito immune pathways, IMD, is important in the defense against the human malaria parasite in three major malaria vectors, *Anopheles gambiae*, *A. stephensi,* and *A. albimanus*. The transcription factor Rel2 is regulated by the IMD pathway and controls the expression of several potent anti-*Plasmodium* immune factors. In this study, we have used germ-line transformation technology to generate transgenic *Anopheles stephensi* mosquitoes with blood meal-inducible expression of *Rel*2 transcripts in both midgut and fat-body tissues. These transgenic mosquitoes were close to completely resistant to human malaria parasites as a result of the timely concerted tissue-specific expression of anti-*Plasmodium* effector genes. Under laboratory conditions, the fitness cost of this genetic modification was minimal, suggesting that this system could be utilized for the development of a malaria control strategy.

## Introduction

One of the world's deadliest diseases, malaria, is caused by protozoan parasites of the genus *Plasmodium,* which are transmitted by *Anopheles* mosquitoes. The absence of effective vaccines and the development of drug-resistant parasites and insecticide-resistant mosquitoes have accentuated the need for novel strategies for malaria control.

The transmission of malaria parasites between human hosts relies on the successful completion of its complex lifecycle in the mosquito vector. The innate immune system of *Anopheles,* this malaria vector's main line of defense against the *Plasmodium* parasite, is engaged at multiple stages of parasite infection [1]–[3]. Among the most potent anti-*Plasmodium* immune factors identified to date are TEP1, APL1, LRRD7, and FBN9, all controlled by the IMD pathway through its transcription factor Rel2 [4]–[12]. Through alternative splicing, the *Rel*2 gene produces a full-length form (*Rel*2-F), which includes the carboxyl-terminal ankyrin (ANK) and death domains, and a shorter form (*Rel*2-S) lacking these inhibition domains, which is constitutively translocated to nucleus, where it actives the transcription of several anti-microbial peptide (AMP) and anti-*Plasmodium* effector genes [9].

We and others have shown that RNAi-based depletion of the negative regulator of Rel2, *Caspar*, results in near complete refractoriness of the three major malaria vectors, *A. gambiae*, *A. stephensi,* and *A. albimanus,* to the human parasite *P. falciparum*, and that over-expression of *Rel*2 in transgenic *Aedes aegypti* mosquitoes results in increased resistance to the avian malaria parasite *P. gallinaceum* [13]. Interestingly, transient activation of *Rel*2 through gene silencing of *Caspar* is associated with a minimal fitness cost in mosquitoes, in terms of their longevity and fecundity under laboratory conditions [4]. These properties of the IMD pathway and its downstream transcription factor Rel2 suggest that, if appropriately manipulated, they could be used for the development of malaria control strategies based on *Plasmodium falciparum*-resistant genetically modified mosquitoes.

Although the innate immune system of mosquitoes has been intensively studied and a plethora of anti-*Plasmodium* effector genes have been discovered over the past two decades, the application of this knowledge to the development of novel malaria control strategies has not been thoroughly investigated. In this study, we have for the first time explored this approach through the development of genetically modified immune-enhanced *Anopheles* mosquitoes that express the active form of the *Rel*2 (*Rel*2-S) transcription factor under the control of the blood meal-inducible carboxylpeptidase (*Cp*) or vitellogenin (*Vg*) promoter [14], [15], in order to target the malaria parasite at its early stages of development (i.e., those associated with the lumen and basal side of the mosquito's midgut tissue). We generated *Cp-Rel*2 and *Vg-Rel*2 transgenic lines, as well as a third hybrid immune-enhanced transgenic line with blood meal-inducible expression of *Rel*2 in both the midgut and fat-body tissue compartments. These three transgenic mosquito lines displayed potent anti-*P. falciparum* activity and provide a unique opportunity to elucidate the spatial and temporal specificities of the mosquito's Rel2-mediated anti-*Plasmodium* defenses. Longevity and fecundity studies of these immune-enhanced transgenic mosquito lines, under laboratory conditions, suggested only a weakly negative impact of transgene expression on these fitness determinants. As a proof of principle, we show for the first time that the mosquito's innate immune system can be used in a genetic engineering approach to develop a control strategy for human malaria.

## Results/Discussion

### Generation and characterization of *Cp*-*Rel*2 and *Vg*-*Rel*2 transgenic mosquitoes

We generated transgenic *Anopheles* mosquito lines that over-expressed an active form of *Rel*2 (*Rel*2S) under the control of the blood meal-inducible midgut- or fat-body- specific promoters. Given the ease of germ line transformation in the *A. stephensi* malaria vector, which also uses the conserved IMD pathway to control infection with *P. falciparum* [4], we chose this species for our analyses. The *A. gambiae Rel*2S was PCR-amplified (primers given in Table S1) and separately ligated to the *A. gambiae* carboxypeptidase A (*AgCp*) and vitellogenin 1 (*AgVg*) promoters in independent constructs, and the terminator sequence of the *A. gambiae* trypsin gene (TryT) was ligated downstream of *Rel*2S (Figure S1A). These two cassettes, *AgCp*-*Rel*2*-*TryT and *AgCp*-*Rel*2*-*TryT, were separately cloned into the piggyBac-based plasmids pBac[3xP3-EGFPafm] and pBac[3xP3-DsRedafm] [16] containing the eye-specific 3xP3 promoter-driven GFP or DsRed as selection markers for the screening of transgenic mosquitoes (Figure S1A).

**Figure 1:**
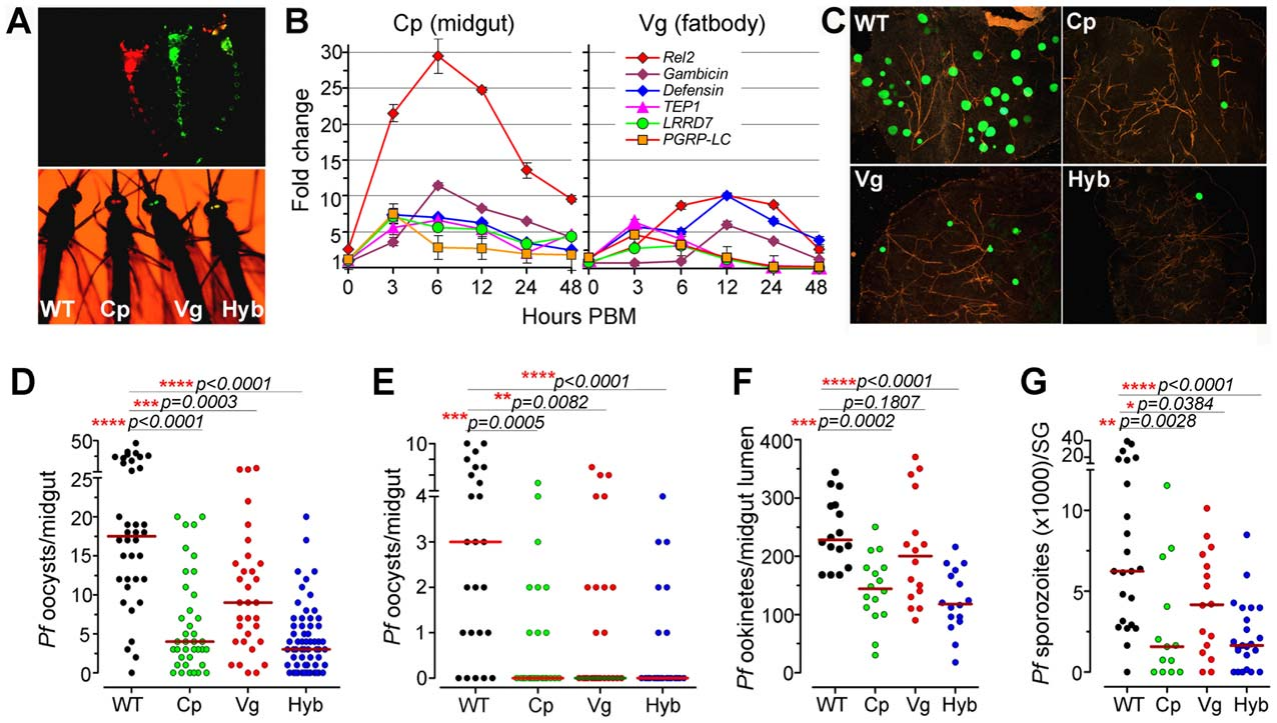
Resistance of genetically engineered *Rel*2-expressing immune-enhanced ***A. stephensi*** **to** ***P. falciparum*** **.** (**A**) Fluorescent images of larvae and adults of the vitellogenin- (Vg, red) and carboxypeptidase- (Cp, green) driven *Rel*2 transgenic lines and hybrid (Hyb, merged into yellow) transgenic line, along with the wt control (WT, non-fluorescent) strain. (**B**) qRT-PCR analysis of *-*fold changes in the expression of the *Rel*2 transgene and effector genes at 0 to 48 h post-blood-meal (PBM), with standard errors shown. Dots represent fold change in expression of the genes of interest (GOI) of the transgenic mosquito tissues compared to that of wt mosquito tissues at the corresponding time PBM. The ribosomal protein S7 gene was used for normalization of the cDNA templates. (**C**) Green fluorescent *P. falciparum* (3D7(GFP)) oocysts in the midguts of WT control, Cp (Cp11), Vg (Vg1), and Hyb mosquitoes at 8 days post-infection (dpi). (**D, E**) *P. falciparum* (NF54) oocyst loads (including zeros) of WT control, homozygous Cp11 and Vg1 and heterozygotes hybrid transgenic mosquitoes at 8 dpi when fed on blood with a standard 0.3% (**D**) and 0.05% (**E**) gametocytemia. (**F**) *P. falciparum* (NF54) ookinete loads in the midgut lumen at 24 h post-infection (hpi). (**G**) *P. falciparum* (NF54) sporozoite loads in the salivary glands at 14 dpi. Assays were performed with at least three biological replicates, and the numbers of parasites from equal numbers of samples (midguts, midgut lumen, or salivary glands) from different replicates were pooled for the dot-plot. Each dot represents the number of oocysts, ookinetes, or sporozoites in an individual midgut, midgut lumen, or salivary gland respectively, and the horizontal lines (red) indicate the median values. Mann-Whitney test determined *p*-values (shown here) and Kruskal-Wallis test was used to calculate *p*-values and determine the significance of oocysts, ookinetes, or sporozoites numbers. Detailed statistical information of infection assays with N, range, prevalence, *p*-values of prevalence determined by Chi-square test, median (with or without zeros), *p*-values of infection intensity (with or without zeros) calculated through Kruskal-Wallis and Mann-Whitney tests, and % decrease of oocysts, ookinetes, or sporozoites loads are presented in Table S3.

By embryo microinjection of these plasmids together with the helper plasmid, we generated 11 *Cp-Rel*2 and 3 *Vg-Rel*2 *A. stephensi* transgenic mosquito lines with stable GFP (Cp lines) and DsRed (Vg lines) eye fluorescence (Figures 1A and S1B). Transgenic mosquitoes were outcrossed with non-transgenic colony *A. stephensi* (referred to as wild-type [wt] controls) for the first four generations to maintain the heterozygous lines. In order to identify the transgenic lines with the most potent anti-*Plasmodium* activity, the fourth and fifth generations of the heterozygous eleven Cp- and three Vg- transgenic mosquito lines were fed on a *P. falciparum* gametocyte culture and the infection intensity was compared to that of wt control colony mosquitoes. This analysis showed that in two consecutive generations of heterozygous mosquitoes, the Cp11 and Vg1 lines displayed the least susceptibility to *P. falciparum* infection, as indicated by oocyst numbers on the midgut's basal side (*p<*0.0001, pooled from two biological replicates from 2 consecutive generations) (Figure S2A-B). Both PCR and Southern hybridization of the genomic DNA from the fourth generation of the transgenic larvae of Cp11 and Vg1 confirmed the transgene insertion (Figure S1C-D). Southern hybridization analysis showed that the genomes of most transgenic lines harbored only one copy of the transgene, while Cp2, Cp6, and Vg2 had two copies (Figure S1D). The exact chromosome locations of the transgene could not be determined because of the lack of an assembled *A. stephensi* genome sequence. The heterozygous transgenic lines except Cp1 displayed different degrees of resistance to *P. falciparum* infection (Figure S2), suggesting that the positional effect of the transgene activity should be taken into consideration when implementing a transgenic mosquito approach for malaria control, given the fact that variations in transgene location and copy number result in different expression of the transgene (effector molecule). The copy number of the transgene did not correlate with the degree of resistance to *P. falciparum* infection (Figures S1 and S2), suggesting that one copy of the *Rel*2 transgene is sufficient to activate the downstream anti-*Plasmodium* effector genes, and that a positional effect on transgene activity plays a more important role in determining resistance. A positional effect could for example influence expression of the transgene *Rel*2, other IMD pathway factors and the downstream effector genes, and thereby result in different degrees of anti-*Plasmodium* activity. One transgene could also influence the expression and activity of another transgene when inserted into the same genome, and the abundance of the transcription factor that activates the transgene will influence its overall transcription. A genetic engineering approach which relies on random integration of the transgene in the genome is therefore useful to determine an optimal integration site for transgene function. A genetic drive system with site-specific recombination, that ensures transgene integration into a defined genomic location, can then be implemented to optimize this system for a malaria control strategy [17]–[20].

After five generations of outcrossing, the heterozygous Cp11 and Vg1 were subsequently interbred to generate homozygous Cp11 and Vg1 lines for further characterization (see details in Materials and Methods section). By crossing homozygous Cp11 female mosquitoes with Vg1 male mosquitoes, we generated a third hybrid transgenic immune-enhanced mosquito line with blood meal-inducible expression of *Rel*2 in both the midgut and fat-body tissue compartments; these three independent lines, homozygous Cp11, Vg1, and the heterozygous hybrid (hereafter referred to as Cp, Vg, and Hyb, respectively), were used throughout this study for the infection assays (Figures 1A and S1B).

### The transgene *AgRel*2 activates IMD pathway-regulated effector genes in *A. stephensi*

To determine whether the *AgRel*2 transgene can activate the expression of downstream AMP and anti-*P. falciparum* effector genes in the *A. stephensi* transgenic mosquitoes, we identified orthologs of the *A. gambiae* immune genes *Gambicin*, *Defensin*, *TEP*1, *LRRD*7, *PGRP-*LC, *APL*1A, and *APL*1C [8], [10], [11], [21]–[23] in the *A. stephensi* genome contig sequence database, then used these sequences for qRT-PCR expression analyses (primer sequences are listed in Table S2). BLAST analyses with *A. stephensi* genome contig sequences suggested that this species only harbors one *APL*1 gene in its genome [5], [7]. A recent study showed that the *APL*1 genes are exceptionally polymorphic and under co-evolution with *TEP*1 [12]. Therefore we designed one pair of primers for the *A. stephensi APL*1 gene, and semi-quantitative RT-PCR of the genes at 12 and 24 hours post blood meal showed that the *APL*1 gene is induced (data not shown). Through qRT-PCR we show that blood meal-inducible over-expression of *Rel*2 (Figure 1B) in the transgenic mosquito midgut and fat-body results in significantly increased transcript abundance of several IMD pathway-regulated anti-microbial and anti-*Plasmodium* genes (*Gambicin*, *Defensin*, *TEP*1, *LRRD*7, *PGRP*-LC) [10], [11], [21], [22], [24] in a temporal and spatial pattern that suggested effective targeting of the malaria parasite. The abundance of the midgut-specific carboxypeptidase-driven *Rel*2 (Cp) transcript peaked at about 6–12 h after blood ingestion, when the parasite is confined to the blood meal in the midgut lumen (Figure 1B) [14], [25]; in contrast, the transcript abundance of the fat-body- specific vitellogenin-driven *Rel*2 (Vg) peaked at about 12–24 h after blood ingestion [13], [15], [26], when the parasite is reaching the basal side of the midgut tissue and it can be more readily targeted by defense mechanisms from the fat-body compartment. However, although *Rel*2 is highly induced in response to a blood meal, its peak expression subsides rapidly after 24 h, suggesting that the induction of an IMD pathway immune response is transient and may not have a strong impact on the transgenic mosquitoes' fitness.

### Impact of *Rel*2 over-expression on *Plasmodium* infection at different sporogonic stages

To assess the anti-*Plasmodium* activity of the three transgenic immune-enhanced mosquito lines, we fed these lines (homozygous Cp11 and Vg1, heterozygous Hyb), along with wt control mosquitoes, on either NF54 or 3D7 (GFP) *P. falciparum* gametocyte cultures or on a *P. berghei* (GFP)*-*infected Swiss Webster mouse. All three transgenic immune-enhanced mosquito lines were significantly less susceptible to the highly virulent *P. falciparum* strain NF54 at the pre-oocyst stage (Figure 1C-G and Table S3). The homozygous Cp and Vg mosquito lines displayed a similar degree of *Plasmodium* resistance compared to the heterozygous mosquitoes (Figures 1D and S2). When the mosquitoes were fed on a laboratory *P. falciparum* gametocyte culture (0.3%) that is known to result in unnaturally high infection intensities and prevalence, the proportion of mosquitoes with at least one oocyst (infection prevalence) decreased significantly in the Cp and Hyb mosquitoes compared to the wt controls (Figure 1D and Table S3; Chi-square test: Cp, *p* = 0.003; Hyb, *p* = 0.0001). The oocyst infection intensity in all three lines was significantly reduced (by 77.1%, 48.6%, and 82.9% in the Cp11, Vg1, and hybrid transgenic mosquitoes, respectively) when compared to the wt controls (Figure 1D and Table S3; *p*<0.001). The hybrid and midgut-specific (*Cp-Rel*2) transgenic lines were more resistant to *Plasmodium* infection than was the fat-body-specific (*Vg-Rel*2) line, suggesting that the parasite is more susceptible to immune attack by carboxypeptidase-driven *Rel*2-mediated activation of the IMD pathway in the midgut, when anti-*Plasmodium* effector genes are expressed at least 6 h earlier than those expressed by the fat-body-driven transgene *Rel*2. We confirmed this hypothesis by showing that the two lines (Cp, Hyb) expressing recombinant *Rel*2 in the midgut tissue were able to decrease the number of ookinete stage parasites prior to invasion of the midgut epithelium (Figure 1F and Table S3), whereas the fat-body-specific *Rel*2 line (*Vg-Rel*2) only displayed an increased capacity to inhibit the post-invasion stages of *Plasmodium* (Figure 1C–E and Table S3). However, since the immune induction of several effector genes was lower in the Vg line compared to the Cp line, we cannot rule out that a more potent driver (promoter) in the fat body (i.e. a different fat-body promoter) might result in an equivalent or stronger protection again *Plasmodium* parasites. These immune defenses resulted in a profound decrease in the infectious sporozoite-stage parasites in the mosquito salivary gland (Figure 1G). The degree of inhibition that we observed was similar to that previously found in other studies utilizing transgenic mosquitoes, but mainly non-human malaria parasites [14], [27]–[29].

The *P. falciparum* laboratory strain used in this study was selected for its ability to produce unnaturally high infection levels averaging as much as 100 oocysts per mosquito in some assays of our study, as compared to a parasite load under natural field conditions that rarely exceeds 2–3 oocysts per mosquito [30]. We therefore hypothesized that the transgenic immune-enhanced mosquito strains would most likely display a greater level of resistance under such natural transmission conditions. To test this hypothesis, we artificially suppressed infections by feeding mosquitoes on *P. falciparum* infected blood at a 6-fold lower gametocytemia (0.05%) than usual (0.3% gametocytemia). This approach produced a refractory state in the immune-enhanced mosquitoes, with a median oocyst count of zero (mean of 0.64) (Figure 1E and Table S3); in contrast, non-transgenic wt control mosquitoes had a median oocyst count of 3 and mean of 3.56. Furthermore, the infection prevalence was 28% in the hybrid line which is significantly lower than the 80% in the wt control mosquitoes (Chi-square test, *p*<0.0001) (Figure 1E and Table S3). However, when mosquitoes with zero oocysts were excluded from the analysis, no differences in the oocyst infection intensities were detected between the three different transgenic lines, corroborating the importance of prevalence when studying *Plasmodium* infection of the mosquito midgut (Table S3). Considering the somewhat unnaturally high levels of infection (exceeding 5 oocysts in 28% of the wt mosquitoes) in this assay, one can expect an even higher proportion of completely resistant transgenic mosquitoes under field conditions, which are characterized by a lower infection rate and prevalence [31]. The level of refractoriness of our immune-enhanced transgenic mosquitoes to the human malaria parasite is similar to that described by Corby-Harris *et al.*, with an unknown mechanistic basis; in that case, the mean oocyst number per mosquito was 0.18, as compared to 3.89 for the wt mosquitoes [32].

Mosquitoes frequently feed multiple times on blood within the ∼12-day time window prior to the sporozoites' invasion of the salivary glands; this repeated feeding would expose the later sporogonic stages of *Plasmodium* to enhanced immune responses in the immune-enhanced transgenic mosquitoes. When we provided a second naïve blood meal 8 days after the parasite-infected blood meal, we saw a non-significant trend (2-fold, Mann-Whitney test, *p* = 0.089) of decreased sporozoite loads in the salivary glands when compared to mosquitoes receiving only one infectious blood meal, suggesting that the transgene *Rel*2-mediated immune response may not implicated in controlling the parasites at the later sporogonic stages (Figure S3). However, the time point of transgene induction upon the second blood meal might not have been optimal for anti-*Plasmodium* defense at this stage, and therefore warrants further analysis.

Transgenic expression of *Rel*2 resulted in a lesser degree of resistance to the rodent malaria parasite *P. berghei,* in only the Cp- and hybrid transgenic lines (Figure S4 and Table S3; Mann-Whitney test: Cp, *p* = 0.0209; Hyb, *p* = 0.0142). Kruskal-Wallis test suggests that overall transgenic mosquitoes showed marginally elevated resistance to rodent malaria parasites (*p* = 0.0363) (Table S3). This result is in agreement with earlier studies showing that the IMD pathway has a more pronounced effect on the human malaria parasite [4], [33].

### The *Rel*2 transgene confers resistance to *P. falciparum* through multiple effector genes

**Figure 2:**
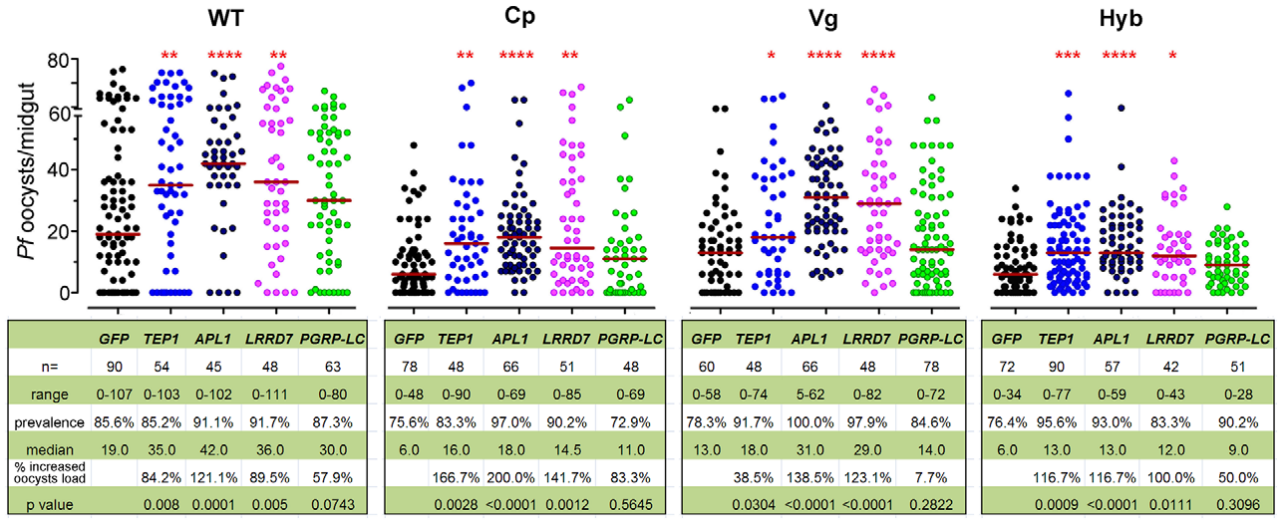
Implication of anti-*Plasmodium* effector genes in refractoriness of immune-enhanced transgenic mosquitoes. Depletion of *TEP*1, *APL*1, *LRRD*7, and *PGRP-*LC through RNAi-mediated gene silencing resulted in changes in the *P. falciparum* oocyst intensity at 8 dpi in the non-transgenic wild type (WT), Cp, Vg, and hybrid transgenic mosquitoes. *GFP dsRNA*-injected mosquitoes served as control. Points indicate the absolute value of oocyst counts in individual mosquitoes, and horizontal red bars in each column represent the median value of oocysts from three replicates. *P-*values were calculated by Mann-Whitney test. (*: *p*<0.05; **: *p*<0.01; ***: *p*<0.001; ****: *p*<0.0001). Detailed statistical information of infection assays with n, range, prevalence, median, *p*-values, and % increased oocysts load are presented in the table beneath the figure as well as in Table S3.

To provide further insight into the mechanism underlying the immune-enhanced mosquitoes' resistance to *P. falciparum,* we show that silencing of the *Rel*2-regulated anti-*Plasmodium* factors, TEP1, APL1, and LRRD7 results in a greater susceptibility to *P. falciparum* infection in transgenic mosquitoes [4], [5], [8], [23]. To confirm the efficiency of gene silencing, qRT-PCR was used to validate transcript depletion prior to feeding on infected blood and at 24 h post blood meal (Table S2). Our data show that each of these anti-*Plasmodium* factors was, at least partially, responsible for the transgene *Rel*2-mediated resistance since their silencing did not result in an increased infection level to the same degree as did their silencing in wt mosquitoes (Figure 2). This observation, taken together with the blood meal-inducible transcription of these genes (Figure 1B), suggests that Rel2-dependent immune activation generates an anti-*Plasmodium* response through a combination of effector molecules that can each attack the parasite independently or synergistically. Our earlier studies have shown that some of these genes engage in independent *Plasmodium* killing mechanisms, while others may act together [4]. *PGRP*-LC gene silencing in the wt mosquitoes resulted in a trend of, but statistically insignificant, increased susceptibility to the *Plasmodium* infection, probably due to a lower silencing efficiency (Table S2) and/or the midgut microflora –elicited basal level of immune activation [33]. The complete lack of effect of *PGRP*-LC gene silencing on the resistance to *Plasmodium* infection in the transgenic mosquitoes may suggest that the modulation of anti-*Plasmodium* effector genes through the *Rel*2 transgene supersedes the modulation of the endogenous IMD pathway by PGRP-LC (Figure 2). The midgut- and fat-body- specific *Rel*2 transgenic mosquitoes generated in this study offer a unique platform for the spatial and temporal dissection of this anti-*Plasmodium* mechanism at molecular and cellular levels.

### Transgene *Rel*2-mediated anti-bacterial defenses

**Figure 3:**
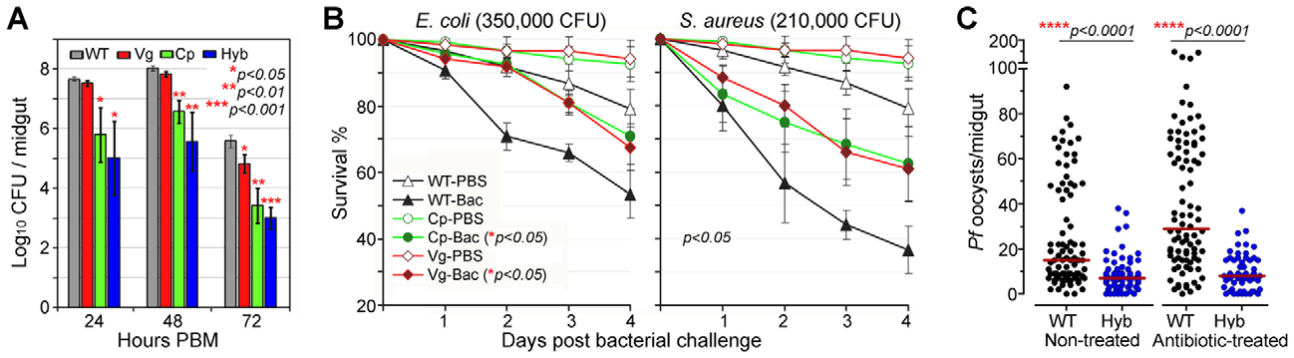
Antibacterial resistance of immune-enhanced transgenic mosquitoes. (**A**) The midgut microbial flora (total bacterial load) of female transgenic and wt control (WT) mosquitoes at 24, 48, and 72 h PBM (mean±SEM). A Student's *t*-test was used to calculate *p*-values and determine significance. (**B**) Survival rates of transgenic mosquitoes upon challenge with either Gram-negative (*E. coli*: 350,000 CFU) or Gram-positive (*S. aureus*: 210,000 CFU) bacteria at 4 dpi. Kaplan-Meier survival analysis with log-rank test was used to determine the *p*-values, and *p*<0.05 indicates significance (detailed Kaplan-Meier survival curves with three biological replicas are presented in Figure S5). (**C**) *P. falciparum* oocyst infection intensities in the septic (non-antibiotic treated) and aseptic (antibiotic-treated) transgenic and WT mosquitoes at 8 dpi. Antibiotic-treated wt mosquitoes became more susceptible to *P. falciparum* infection than did non-antibiotic-treated wt mosquitoes, while oocyst intensities in transgenic hybrid mosquitoes showed no change. At least three biological replicates are included, with each dot representing the number of oocysts in an individual midgut, and the horizontal lines (red) indicating the median values. *P*-values were calculated by a Mann-Whitney test.

In nature, mosquitoes are exposed to a variety of microbes, both via injury and from their normal intestinal flora, and the IMD pathway was originally identified as the insect's main defense against Gram-negative bacteria [34], [35]. The immune-enhanced transgenic mosquitoes that express *Rel*2 in their midgut tissue displayed elevated resistance to the microbes of their midgut flora, which mainly consist of Gram-negative species (Figure 3A), corroborating findings from previous studies[36], [37]. At 24 to 72 h post-blood meal (PBM), the bacterial loads in the Cp11 and Hyb lines were significantly lower than those of the wt controls, suggesting that *Cp* promoter-driven over-expression of *Rel*2 in the midgut mediated a robust expression of AMPs, which were acting against the midgut bacteria even when bacterial loads peaked at 48 h PBM (Student's *t*-test, *p*<0.01). Only the LB culturable microbial flora was assessed which was composed of the dominant bacteria *Serratia* spp. and *Cryseobacterium* spp. from laboratory-reared female mosquito midguts [36], [37].

Compared to those expressing *Rel*2 only in the midgut, mosquitoes that expressed *Rel*2 in both the midgut and fat-body tissue displayed significantly greater resistance to systemic challenge (thoracic microbial injection) with the Gram-negative bacterium *E. coli* (Kaplan-Meier survival analysis, *p*<0.05), as well as with the Gram-positive bacterium *S. aureus* (Kaplan-Meier survival analysis, *p*<0.05) (Figures 3B and S5) (detailed Kaplan-Meier survival curves are presented in Figure S5 with three biological replicates shown). Interestingly, increased survival can also be observed in PBS-injected transgenic mosquitoes when compared to the wt parental mosquitoes which might be contributed from the introduction of bacteria through the injection process. The fitness trade-offs between a potentially costly bloodmeal-inducible immune response and the predicted favorable effect of an increased resistance to certain bacteria will be interesting to address in greater detail in future studies. However, one could also argue that a reduction of the microflora through recombinant *Rel*2 expression could counteract the anti-*Plasmodium* activity of certain natural midgut bacteria [38], and further studies will be necessary to examine this possibility.

We and others have shown that the mosquito's midgut microbioal flora is responsible for a basal activation of the IMD pathway-regulated immune genes, which in turn influence resistance to *Plasmodium* [24], [37]. To determine whether the presence or absence of midgut bacteria would influence the resistance of transgenic immune-enhanced mosquitoes to *Plasmodium* infection, we performed identical infection assays with wt and hybrid transgenic mosquitoes under septic (non-antibiotic treated) and antibiotic-treated aseptic conditions. While removal of the majority of the midgut bacterial flora (either LB culturable or Giemsa stainable) through antibiotic treatment resulted in an increased susceptibility to *Plasmodium* infection in the wt control mosquitoes, as shown earlier, the resistance of the transgenic immune-enhanced line was not affected (Figure 3C), suggesting that the recombinant *Rel*2 prevails over the activity of the bacteria-inducible endogenous *Rel*2. The dominance of the *Rel*2 transgene upon blood meal induction was also indicated by our experiments in which depletion of *PGRP*-LC, which acts upstream of the IMD pathway, had no effect on resistance to *Plasmodium* infection (Figure 2).

**Figure 4:**
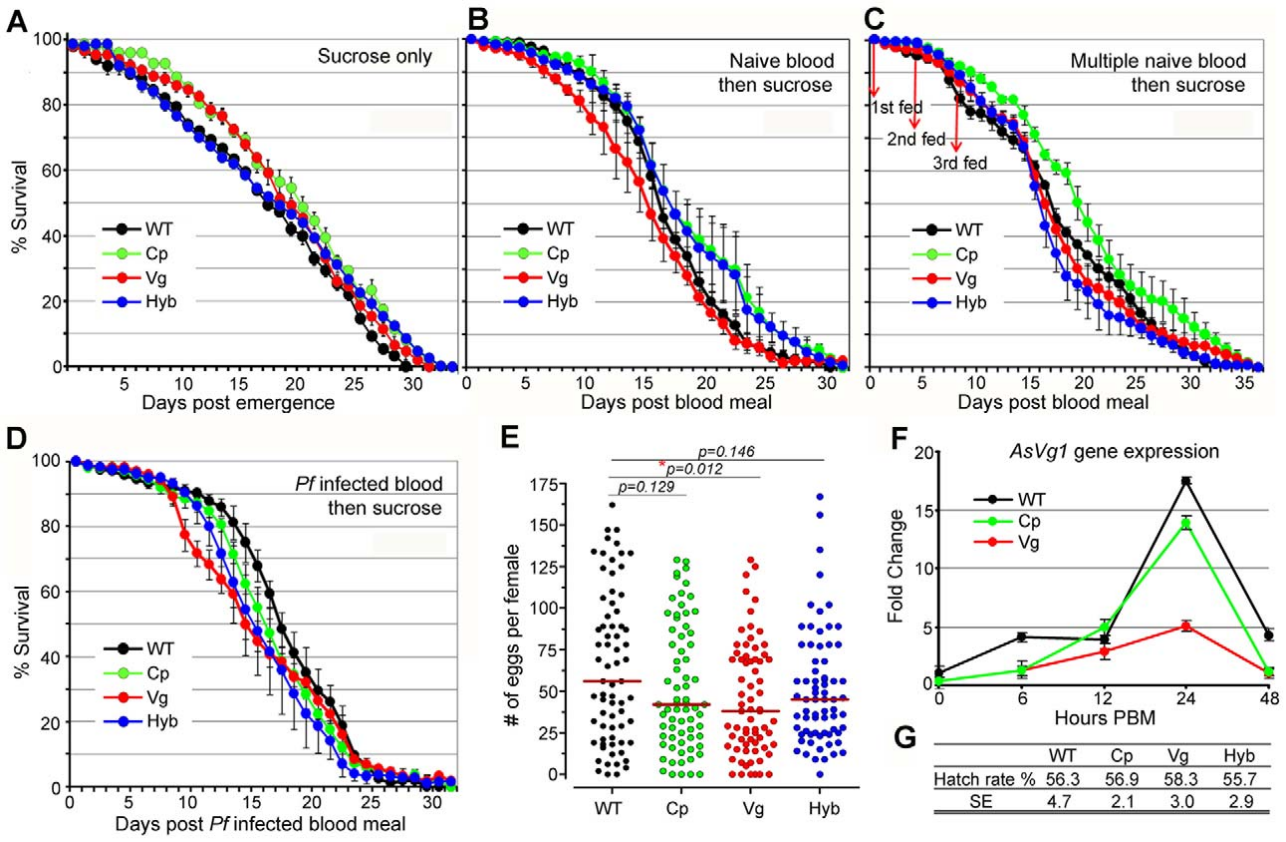
Fitness assessment of immune-enhanced transgenic mosquitoes. (**A–D**) Life spans of transgenic and wt mosquitoes maintained on 10% sucrose solution (**A**); or mosquitoes fed on a single naïve human blood 5 d post emergence and then maintained on sugar solution (**B**); or mosquitoes that were provided 3 naïve blood meals (days 0, 4, 8) and then maintained on sugar solution (**C**); or mosquitoes that were provided a *Pf*-infected blood meal (0.3% gametocytemia) and then maintained on sugar solution (**D**). The mean values from three replicates are shown, with the standard error bars. Survival rates were analyzed by Kaplan-Meier survival analysis with Wilcoxon test to determine the significance, and detailed statistical information is shown in Table S4. (**E**) Eggs laid by female transgenic and wt mosquitoes from three biological replicates: Each dot represents the eggs laid by an individual female after a single blood meal. The median values (red horizontal bars) are shown. *P*-values were calculated with a Mann-Whitney test. (**F**) -Fold change in the expression of the *A. stephensi* endogenous vitellogenin 1 (*AsVg1*) gene, at 6, 12, 24, and 48 h PBM (mean±SEM) compared to that of the non-fed mosquitoes. The cDNA templates were normalized using the *AsS7* gene. (**G**) Hatch rates indicate the average percentage of eggs giving rise to 1st instar larvae, as determined by three biological replicates. Mean values for hatch rates and standard errors (SE) of replicates are indicated.

*Rel*2 immune-enhanced transgenic mosquitoes display a marginal decrease in longevity and fecundity under laboratory rearing conditions. Immune responses are generally known to be associated with fitness trade-offs [39]-[41], and these associations could compromise the implementation of a immune-enhanced transgenic mosquito for malaria control. When mosquitoes were maintained on a 10% sugar solution in the absence of a blood meal that would induce the expression of the *Rel*2 transgene, the longevity of the immune-enhanced mosquito strains did not differ from that of the parental wt control strain under laboratory rearing conditions (Figure 4A and Table S4) (detailed statistical analysis for three biological replicates are listed in Table S4). Exposure of immune-enhanced transgenic mosquitoes to a single naïve blood meal resulted in an increased longevity of one and two cohorts of Cp and hybrid mosquitoes, respectively (Figure 4B and Table S4). There was an insignificant change in the survival of the transgenic mosquitoes after multiple naïve blood meals, with only one cohort of Cp transgenic mosquitoes possessing a longer life span (Figure 4C and Table S4). After a single *Pf*-infected blood meal, hybrid mosquitoes showed a significant reduction in longevity in three independent assays (Kaplan-Meier survival analysis, *p*<0.05), while one cohort of Cp and Vg mosquitoes also displayed a significantly shorter life span (Figure 4D and Table S4). Fecundity, as a measure of egg-laying capacity, was only slightly lower in the transgenic fat-body *Rel*2-expressing strain (Figure 4E), possibly because of a decreased expression of the egg yolk protein vitellogenin during IMD pathway activation (Figure 4F), as also shown by an earlier study [42], and a likely competition between the transgenic and endogenous *Vg* promoters. Furthermore, the egg hatch rate was unaffected by the transgene in the case of all three immune-enhanced mosquito strains (Figure 4G).

Our data are consistent with earlier studies in *D. melanogaster* which showed that a transient activation of the IMD pathway, through overexpression of a *PGRP* gene (*PGRP*-LE), did not influence fly fitness as a measure of longevity under abundant and limited food provision and the ability of the flies to jump [43]. These studies also showed that a constitutive activation of this pathway did result in a significant decrease on longevity. However, the fitness impact of transgene effector molecule expression needs to be addressed in a greater detail prior to the implementation of a malaria control strategy. The fitness parameters addressed in this study are limited and do therefore not fully predict the overall fitness of the immune-enhanced transgenic mosquitoes. Other parameters, such as fertility, larval biomass productivity, developmental rate, adult emergence, male ratio, and mating competitiveness including life table analysis and cage experiments, as suggested in [44]–[46], will be addressed in future studies. Nevertheless, our fitness assays suggest that the fitness loss associated with expression of the transgene *Rel*2 is unlikely to impair the spread of the immune-enhancement trait in natural mosquito populations when an effective genetic drive system that can overcome fitness disadvantages is employed [17], [18]. For example, the *Drosophila Medea* elements are predicted to convert an entire population into element-bearing heterozygotes and homozygotes even at a 15% element-associated fitness cost, when these elements are introduced into the population at relatively high frequencies [17].

In conclusion, we have genetically engineered *A. stephensi* mosquitoes to spatially and temporally express the *Rel*2 transgene in both midgut and fat-body tissues after a blood meal; thereby activating robust expression of several AMPs and anti-*Plasmodium* effector genes. We have investigated the spatial and temporal impact of Rel2-mediated innate immune responses on *P. falciparum* development and found that over-expression of several anti-*Plasmodium* effector genes (*TEP*1, *APL*1, *LRRD*7) in these *Rel*2 transgenic mosquitoes contributed to the *Plasmodium*-refractory phenotype. Over-expression of *Rel*2 in both the midgut and fat-body tissue in these mosquitoes limited the proliferation of the midgut microbial flora, and increased the mosquitoes' resistance to systemic challenge with Gram-negative bacteria. By using single and hybrid transgenic lines, we also addressed for the first time the fitness implications of the mosquito's Rel2-mediated innate immune responses in various tissues in a transgenic context and demonstrated that transient activation of *Rel*2 in the gut tissue has only a minimal impact on relevant fitness parameters under laboratory conditions, and this finding is consistent with an earlier study using *Drosophila* [43]. At this stage we cannot exclude the possibility of some parasites developing resistance to this blocking mechanism, and the fact that some of our transgenic mosquitoes possessed sporozoites in their salivary glands corroborates the need for further studies of this defense system. A different spatial-temporal transgene induction profile or the combination of multiple anti-*Plasmodium* transgenes may render mosquitoes completely parasite-proof.

Our current and previous studies have shown that a transgenic *Rel*2-mediated innate immune response fulfills several criteria required for an anti-*Plasmodium* effector system that could be used for malaria control. First, we have shown that genetically modified immune-enhanced mosquitoes activate multiple anti-*Plasmodium* factors that are likely to act independently or synergistically against *Plasmodium,* thereby decreasing the possibility for the development of resistance by the parasite [4]–[6], [8], [9], [23]. It is, however, important to note that it remains to be demonstrated that this approach will provide protection in the field, with its enormous genetic diversity of wild *Plasmodium* populations. Second, *Rel*2 confers resistance to *P. falciparum* in three independent and evolutionary divergent malaria vector species, as well as to the bird malaria species *P. gallinaceum* in *Aedes aegypti*, potentially rendering this approach feasible for application to the ∼40 different *Anopheline* vectors of malaria [4], [13]. Third, the fitness effect of transient *Rel*2 activation in the midgut tissue, as measured by longevity and fecundity under laboratory conditions, would not impair the spread of the transgene by a powerful genetic drive system [17], [18]. Fourth, our approach did not involve the introduction of a foreign recombinant gene, but only an enhancement of the mosquito's innate immune system through over-expression of its own *Rel*2 transcription factor through its own carboxypeptidae and vitellogenin promoters, thereby decreasing the possibility of unexpected adverse effects relating to expression of a heterologous protein. A plausible future scenario could involve the spread of a *Rel*2 transgene through a powerful genetic drive system that can overcome the fitness cost of transgene expression, thereby conferring enhanced-immune properties on existing natural malaria vector populations. This approach has the advantage of being logistically simple and self-propagating as well as environmentally friendly, since it does not eliminate the mosquito from its ecologic niche or involve chemical insecticide or drug treatments.

In sum, we have for the first time shown, as a proof of principle, that the mosquito's innate immune system has the potential to be used in a genetic engineering approach to block the transmission of human malaria parasites.

## Materials and Methods

### Ethics statement

This study was carried out in strict accordance with the recommendations in the Guide for the Care and Use of Laboratory Animals of the National Institutes of Health. The protocol was approved by the Animal Care and Use Committee of the Johns Hopkins University (Permit Number: M006H300). Commercial anonymous human blood was used for parasite cultures and mosquito feeding and informed consent was therefore not applicable. The Johns Hopkins School of Public Health Ethics Committee has approved this protocol.

### Mosquito rearing and antibiotic treatments

*A. stephensi* Liston strain mosquitoes were maintained under laboratory conditions at 27°C and 80% humidity with a 12 h day-night cycle. Larvae were reared on cat food pellets and ground fish food supplement. Adult mosquitoes were maintained on 10% sucrose and fed on mouse blood (mice were anesthetized with ketamine) for egg production. The antibiotic treatment of the mosquitoes was performed according to [37] to obtain mosquitoes with an eliminated LB culturable midgut microbial flora. In brief, a single cohort of adult female mosquitoes was collected immediately after eclosion and placed in a sterile environment. In the first consecutive 4-day period after eclosion, adult female mosquitoes were reared on a fresh filter-sterilized 10% sucrose solution containing 15 µg gentamicin sulfate (Sigma) and 10 units/10 µg of penicillin-streptomycin (Invitrogen) per ml. The efficacy of removal of the majority of bacteria in the gut of antibiotic-treated mosquitoes was confirmed by plating gut homogenate on LB-agar plates and through Giemsa staining of midgut homogenates as described in [37]. One day prior to feeding on *P. falciparum-*infected blood, mosquitoes were provided with fresh filter-sterilized sugar without antibiotics. Antibiotic treatment was applied again after feeding until dissection.

### Transformation vector constructs

For the mosquito germ-line transformation, we used the pBac[3xP3-EGFPafm] and pBac[3xP3-DsRedafm] transformation vectors containing an eye-specific promoter (3xP3) in front of the TATA box, as described in [16]. The active form (1840 bp) of the *A. gambiae Rel*2 (*Rel*2S) lacking the ankyrin repeats and death domains [9] was first cloned into the pBluescript vector at the *Eco*RI site (Stratagene). The *Rel*2 primers used for amplifying the PCR product from *A. gambiae* cDNA were: 5′-GCAGTGGTCAGTGTTGGAGAG-3′ (forward primer) and 5′-TTCCGAGTTACAGGGGAA GTC-3′ (reverse primer). A 392-bp DNA fragment of the putative terminator region of trypsin was obtained by PCR from the vector pENTR-carboxypeptidase P-antryp1T (kindly provided by Dr. Yoshida) [28] and was cloned downstream of *Rel*2 at the *Spe*I/*Not*I site of the pBluescript vector. The *A. gambiae* vitellogenin 1 promoter (1800 bp) was obtained from *A. gambiae* gDNA, and the carboxypeptidase A promoter (2311 bp) was obtained by PCR from the vector pENTR-carboxypeptidase P-antryp1T [28] (primer sequences listed in Table S1). Both promoters were separately cloned in-frame upstream of the *Rel*2 into the pBluescript vector. The *AgVg*-*Rel*2S-TryT and *AgCp*-*Rel*2S-TryT cassettes were individually digested and cleaved from the *Fse*I site in the pBluescript vector, then cloned into the *Fse*I sites of the pBac[3xP3-DsRedafm] or the pBac[3xP3-EGFPafm] vector, respectively. These two resulting plasmids, pBac-DsRed[*AgVg*-*Rel*2-TryT] and pBac-EGFP[*AgCp*-*Rel*2-TryT], were then used for injection into the *A. stephensi* embryos (Figure S1).

### Generation of transgenic mosquitoes

For the germ-line transformation, the donor (*Rel*2-containing plasmids) and helper plasmid phsp-pBac were prepared using the Qiagen Endofree Maxi Prep kit and re-suspended in 1x microinjection buffer according to published methods [14], [47]–[48]. A mixture of 0.25 µg/µl of the *Rel*2 plasmid and 0.25 µg/µl of the helper plasmid DNA were injected into *A. stephensi* embryos using the Eppendorf Transjector 5246 and Quartz needles according to established protocols [47], [49]. To generate *Cp*-*Rel*2-*GFP* transgenic mosquitoes, 1528 *A. stephensi* eggs were injected and the 244 hatched larval survivors were screened for transient expression of the GFP marker (green eyes). Approximately 38% showed transient expression of *GFP*, and all 208 survived pupae were sexed first (123 females and 85 males) and then organized into about 20 families (12 female and 8 male families). These different groups of mosquitoes were outcrossed to wild-type mosquitoes independently with about 9 survived G_0_ female mosquitoes crossed with 3 wt male mosquitoes (3∶1), while 9 survived G_0_ male mosquitoes were crossed with 45 wt female mosquitoes (1∶5). The F_1_ progeny was examined for green fluorescent glowing eyes at both larval and adult stages. Eleven *eGFP*-expressing mosquitoes from 20 different groups were collected and outcrossed to the wt mosquitoes (in each generation 10 female transgenics were outcrossed with about 5 wt non-transgenic parental male mosquitoes) for consecutive 4 generations which finally gave rise to 11 independent trangenic lines (named from Cp1 to Cp11). For the *Vg*-*Rel*2-*DsRed* transgenic line, approximately 1500 embryos were injected, and the 210 hatched larval survivors were screened for transient expression of the DsRed marker (red eyes). Similarly, these survived mosquitoes were divided into about 15 families which were outcrossed with wt mosquitoes in the individual groups which finally gave rise of three independent *Vg-Rel*2*-DsRed* lines (named Vg1, Vg2 and Vg3). Among all these lines, Cp11 and Vg1 were selected for further studies. After five generations of outcrossing, homozygous Cp11 and Vg1 lines were generated for further characterization. After 5 generations of outcrossing, heterozygous Cp11 or Vg1 mosquitoes were subsequently kept interbreeding for about 5 generations to generate homozygous lines of Cp or Vg for further characterization, in addition to producing a hybrid line. In each generation, about 20 female transgenic Cp or Vg mosquitoes which showed the strongest fluorescence in eyes (either green for Cp or red for Vg) were collected and interbred with about 7 male Cp or Vg with similar fluorescent intensities in the eyes. The offspring were collected and kept interbreeding in the same manner for another 4 generations until the homozygosity was determined, when outcrossing of homozygous female mosquitoes with wt male mosquitoes produced not a single offspring mosquito with wt phenotype. Homozygous Cp or Vg mosquitoes were subsequently interbreeding and used for *Plasmodium* infection assays. Female homozygote Cp11 was crossed with male Vg1 to generate a third hybrid transgenic line (Hyb, with both green and red eyes when viewed with appropriate fluorescent filters). After screening of hybrid mosquitoes for more than five generations (each generation of transgenic larvae were screened for both green and red eyes), we were unable to obtain homozygous Hyb mosquitoes, probably due to an unknown mechanism which needs further characterization. Finally in each generation there were always about 25% larvae with only one color of fluorescence in the eyes, and the hybrid line we used in this study is a mixture of homozygotes and heterozygotes which is thereafter referred as “heterozygote”. Further work will be required to provide potential explanations for this observation. To assure that the hybrid line harbors both *Cp-Rel*2 and *Vg-Rel*2, hybrid mosquitoes were screened every generation for the assays. The homozygous Cp11 and Vg1 mosquitoes, heterozygous hybrid mosquitoes (Cp11, Vg1, and Hyb) were used for all the assays performed after the initial *P. falciparum* infection screen and were referred to as the Cp-, Vg- and Hyb- lines. The wt control (parental) colony mosquitoes were reared in parallel to the transgenic mosquitoes for all the infection assays.

### Genomic DNA extraction and Southern hybridization

Genomic DNA (*g*DNA) was extracted from wild type, Cp1 to Cp11, Vg1 to Vg3 strains by using Qiagen DNeasy Tissue kit. About 0.1 µg of *g*DNA was used for PCR to confirm the insertion of transgene (*Rel*2), GFP or DsRed in the transgenic mosquitoes by using the primers (Rel2-veri, GFP-veri, DsRed-veri) listed in Table S1. Southern hybridization analysis was done as described in [13] with some modifications. Briefly, for each mosquito strain tested, about 15 µg *g*DNA was digested by using *EcoR*I, *Hind*III (New England Biolabs). After precipitation of the digested products, the DNA pellets were resuspended in 20 µl of TE buffer, and loaded on a 0.8% agarose gel which ran about 4 hours (or overnight) to separate the DNA fragments. After alkaline transfer of DNA to a positive charged nylon membrane (Roche), the membrane was hybridized with the DNA probes of *Rel*2, right *piggyBac* arm for *EcoR*I-restricted DNA, and *GFP*, *DsRed* region for *Hind*III-digested DNA. The probe labeling and hybridization were done by using DIG High Prime DNA Labeling and Detection Kit (Roche). Detailed probe sequence regions are presented in Figure S1A.

### RNA isolation, quantitative real-time PCR (qRT-PCR) and RNA intereference (RNAi)-mediated gene silencing

RNA was extracted and quantified (in triplicate samples) in different tissues (whole mosquito, gut, and fat-body) using RNeasy kit (Qiagen), and cDNA was prepared using oligo(dT) primer according to standard methodology using Invitrogen Superscript III reverse transcriptase. The quantitative real-time PCR (qRT-PCR) and RNAi gene-silencing assays were done according to [10] with primers from Table S2, and the ribosomal protein S7 gene was used for normalization of the cDNA templates. The -fold change in the gene expression and the gene silencing efficiency (from RNAi assays) were calculated according to the standard E^ΔΔCt^ method [50] when both primer efficiencies between GOI (gene of interest) and S7 gene are equal. The primer efficiencies were determined as described in [50].

Several *A. stephensi* immune genes (*TEP*1, *LRRD*7, *APL*1, and *PGRP-*LC) were screened for anti-*Plasmodium* defense activity and potential immuno-regulatory function using RNA interference (RNAi) in the wild-type, Cp-, Vg-, and Hyb- lines. For these assays, the gene mRNA was selectively depleted from the adult female mosquitoes using established RNAi methodology [22]. The *dsRNA* injection assay of different genes was repeated at least three times with at least 80 mosquitoes in each experiment; the *GFP dsRNA*-injected mosquitoes served as controls. The RNAi gene silencing efficiencies were determined for all WT, Cp11, Vg1, and hybrid lines at 3 d post *dsRNA* injection for all 4 genes tested compared to *dsGFP* injected control mosquitoes. Considering that the transgene *Rel*2 and the effector genes are strongly induced post blood meal in the transgenic mosquitoes, therefore we also checked the gene silencing efficiencies at 24 h post blood feeding (pbf) for *TEP*1, *LRRD*7, and *PGRP-*LC genes (Table S2). The primers used for *dsRNA* synthesis and silencing verification, along with the gene silencing efficiencies, are presented in Table S2.

### P. falciparum and P. berghei infection assays

To determine the anti-*Plasmodium* activities, the transgenic and wild-type mosquitoes were fed on either NF54 or 3D7 (GFP) *P. falciparum* gametocyte cultures (provided by the Johns Hopkins Malaria Institute Core Facility, Sanaria, and Dr. Sinden) [51] through artificial membranes at 37°C or on a *P. berghei* ANKA (GFP)*-*infected Swiss Webster mouse (at 19°C) [10], [52]. The NF54 *P. falciparum* clone (Sanaria) was used throughout the study except for Figure 1C where the 3D7 (GFP) clone was used to generate a representative view of comparing *P. falciparum* oocysts infection intensities in different transgenic and wt mosquitoes. The adult mosquitoes were starved for 8–10 h prior to feeding to ensure engorgement. To determine oocyst numbers, unfed mosquitoes were removed after 24 h, and the rest were incubated for a further 7 days at 27°C or 13 days at 19°C for *P. falciparum* and *P. berghei*, respectively. Midguts were dissected out in PBS, stained with 0.2% mercurochrome, and examined using a light-contrast microscope (Olympus). At least three biological replicas were performed for each experiment, and oocyst numbers from equal numbers of midguts from different replicates were pooled for producing the dot-plot through GraphPad Prism5 software.

Ookinete counting in the mosquito guts and lumen was done according to established methodology [37], [38], with minor modifications. The guts, including the entire bloodmeal contents, were placed in Corning 96-well plates with 40 µl of sterile PBS and individually homogenized by repeated pipetting; 10 µl of this homogenate was then spotted onto Teflon-printed microwell glass slides (VWR International) previously coated with 3-aminopropyltriethoxysilane (APES) according to the supplier's instructions (Sigma). The sample slides were then air-dried and fixed with methanol, then stained with Giemsa stain for 45 min and analyzed under a Nikon E800 microscope. The total number of ookinetes in each spotted sample was counted, and average values for the densities of the ookinetes were calculated from at least two biological replicates. These average values were then multiplied by the dilution factor of the sample (1 in 4) to give an estimate of the total number of each malarial parasite stage that was present within the entire blood meal.

A method described in [38] was used to determine the sporozoite loads in the salivary glands of the infected mosquitoes, salivary glands were dissected, and individual glands were placed in Eppendorf tubes with 120 µl of PBS, then homogenized (on ice). The homogenate was centrifuged at 8,000 rpm for 10 min, followed by the removal of approximately 90 µl of supernatant. The sporozoites were resuspended in the final 30 µl of PBS, and 10 µl of this suspension was placed in a Nuebauer counting chamber and counted after 10 min using a Leica phase-contrast microscope at 400x magnification.

The dot plots of the ookinete, oocyst, and sporozoite numbers in each gut lumen, gut epithelium, and salivary gland, respectively, for each treatment were generated using GraphPad Prism5 software, along with the median value indicated. For the statistical analysis, we first tested statistical differences between different replicates within the same treatment as described in [5], then pooled the replicates from the equal number of samples (randomized before pooling) for the dot plots when no differences between replicates were detected. Since the *Plasmodium* infection prevalence did not show great variations between the wt and transgenic mosquitoes when at the higher infection levels, we included midguts with zero oocyst in our analyses as described in [4], [37], [53]. *P*-values were calculated through non-parametric Mann-Whitney test or Kruskal-Wallis (KW) ANOVA on ranks and used to determine the significance of differential infection levels as described in [5], [37]. To gain a more detailed picture of prevalence and infection intensity, we separated these two attributes into two data sets for the statistical analysis as presented in Table S3, where prevalence of oocysts was analyzed for each replicate independently and were then pooled and analyzed. Significant differences of prevalence were determined through a Wald Chi-square test as described in [5], [32]. Median parasites numbers and range of all mosquitoes that were infected are also presented in Table S3.

### Bacterial challenge survival assay and characterization of proliferated midgut microbial flora

Isolation and colony-forming unit (CFU) enumeration of endogenous gut bacteria from WT, Cp-, Vg-, and Hyb- female mosquitoes were performed as described previously, with modifications [37], [54]. Age-matched mosquitoes were provided with a blood meal, and engorged mosquitoes were separated and kept under standard insectary conditions for the remainder of the experiment. On the day of the blood-feed, unfed mosquitoes from each cohort were selected for analysis, while blood-fed mosquitoes were used for all analyses post-blood meal (PBM). Each mosquito was surface-sterilized by washing twice with 70% ethanol and rinsing in sterile PBS; to determine the efficacy of sterilization, mosquitoes were placed on LB agar prior to dissection. The gut was dissected with sterile forceps and placed in sterile PBS. Ten-fold serially diluted midgut homogenates were plated on LB agar and incubated at ambient temperature for 4 days. Each experiment was performed using an individual gut, and results are representative of eight individual experiments.

Survival assays conducted following bacterial challenges of *Rel*2 transgenic mosquitoes were conducted as an adaptation of an established protocol [10], [13]. Vg-, Cp-, and wt 4-d-old females were fed on anesthetized mice and challenged with bacteria at 3–6 h PBM. An overnight LB culture of bacteria were further washed and re-suspended in PBS; 69 nl of the bacterial suspension was injected into the mosquito's thorax with a nano-injector (Nanoject, Drummond) at 350,000 CFU (for *E. coli*) and 210,000 CFU *(*for *S. aureus*) per mosquito. For negative controls, 69 nl of PBS was injected into WT, Vg, or Cp mosquitoes. Dead mosquitoes were counted and removed from cages daily over a 4 d period after challenge with bacteria. Forty blood-fed females were used for each group of injected mosquitoes, and at least three replicates were performed for all experiments. The significance of transgene *Rel*2 expression for the mosquitoes' susceptibility to bacterial infection was determined using Kaplan-Meier survival analysis with log-rank test used for significance evaluation with GraphPad Prism5 software as described in [54] (Kaplan-Meier survival curves are presented in Figure S5).

### Longevity, fecundity, and egg hatchability assays

Before these experiments were carried out, the three transgenic lines were outcrossed with non-transgenic wild-type mosquitoes for at least three generations to ensure a genetic background similar to that of the wt control mosquitoes, and heterozygous Cp, Vg, and hybrid lines were used for the assays. Crosses between heterozygous transgenic (Cp and Vg) and wt parental non-transgenic mosquitoes produced a 50/50 ratio of transgenic to wt siblings. Meanwhile, the non-transgenic wild type colony mosquitoes used for outcrossing were maintained under exactly the same conditions as the transgenic mosquitoes. These offspring were then used for the following assays: For longevity assays with mosquitoes maintained on sucrose solution only, approximately 50 to 80 4-d-old adult female wild-type and transgenic mosquitoes (Cp, Vg, and Hyb) were kept in a wax-lined cardboard cup at 27°C with 70% humidity and maintained on a sterile 10% sucrose solution. The mosquitoes' survival rate was also monitored by providing a single naïve human blood meal to 5-d-old mosquitoes that were then maintained on 10% sucrose solution, or by providing three naïve human blood meals to 5-d-old mosquitoes (at day 0, 4, 8) followed by maintenance on a 10% sucrose solution until day 30; after each feeding, the unfed mosquitoes were identified, recorded, and removed. At day 0 the 100% survival refers to those mosquitoes fed for the first time, for the second and the third blood-feeding, the mosquitoes which did not feed were removed and censored in the survival analysis. Blood-feeding propensity of the transgenic lines did not show difference from that of wt mosquitoes, therefore not shown here. Alternatively, 5-d-old mosquitoes were provided with a *Pf*-infected blood meal (0.3% gametocytemia) and then maintained on a 10% sucrose solution, and the infection level was then confirmed by counting the oocysts at 8 days post-infection from 10 mosquitoes for each treatment. Three independent experiments were performed, and all cohorts were monitored daily for survival; the dead mosquitoes were removed each day. Monitoring continued until all mosquitoes had perished. The survival percentage represents the mean survival percentage for all three biological replicates of 50 to 80 mosquitoes each (the exact numbers of mosquitoes were recorded in Table S4) as described in [4]. Statistical significance was determined by Kaplan-Meier survival analysis with GraphPad Prism5 software, and *p*-values were determined by Wilcoxon test as described in [32]. For the fecundity assay, approximately 50 4-d-old adult female wt and transgenic mosquitoes (Cp, Vg, and Hyb) were allowed to feed on human blood through an artificial membrane feeder for 30 min. The fed mosquitoes were transferred to individual wax-lined cardboard cups (one mosquito per cup) outfitted with cotton soaked in 10% sucrose solution and an oviposition cup filled with water and lined with the filter paper. Individual chambers were incubated under normal rearing conditions. Eggs oviposited on filter paper were counted after 2 d using light microscopy. Female mosquitoes that did not produce eggs on day 2 were maintained and re-examined on day 3. After each count, eggs were submerged in a standard larval pan for rearing according to standard methods. First instar larvae were counted and removed from the larval pan daily to determine the larval hatch rate. The fecundity and larval hatch-rate assays were performed for three consecutive generations (three biological replicates), and the number of eggs laid by each female and their hatch rate were pooled to calculate the median value. Statistical significance was determined using the Mann-Whitney test.

## Supporting Information

Figure S1 **Generation of** ***Rel2*** **transgenic lines in** ***A. stephensi*** **.** (**A**) Schematic representation of the pBac-EGFP[*AgCp*-*Rel*2-TryT] and pBac-DsRed[*AgVg-Rel*2-TryT] transformation plasmids used for the germline transformation of *A. stephensi*. The restriction sites used for DNA digestion for Southern hybridization are indicated; the thick horizontal black lines indicate the probes (*GFP*, *DsRed*, *piggybac* Right arm) regions. Double-headed arrows indicate the sizes of the inserts of *AgCp-Rel*2 and *AgVg-Rel*2 for making the constructs. The sizes of each region of the corresponding constructs are indicated by the numbers above the schematic map. (**B**) Bright-field and fluorescent images of larvae and adults of the Cp, Vg, and Hyb transgenic lines. The control wild-type (WT) is also shown. (**C**) PCR confirmation and Southern blot analysis of transgene integration. In each row, column 1 shows the transgenic line DNA, column 2 the non-transgenic control WT DNA, and column 3 the plasmid DNA used in germ-line transformation. The top row is the PCR amplification of the fluorescent marker (GFP for Cp, and DsRed for Vg), and the middle row is the PCR amplification of the *Rel*2 transgene. The lower row is a Southern blot analysis of the *Rel*2 transgene on *Eco*RI-digested genomic DNA (*g*DNA) of the transgenic Cp11 and Vg1 lines, WT, and control plasmid. (D) Southern blot analysis of the genomic DNA isolated from the representative Cp and Vg transgenic strains and the wild-type control strain. *Hind*III and *EcoR*I were used for the *g*DNA digestion, probes from PCR products of *GFP*, *DsRed*, and *piggyBac* right arm were used for the Southern hybridization. WT: wild-type control parental strain; Cp: Cp lines; Vg: Vg lines.
(TIF)

Figure S2 ***P. falciparum*** **(NF54) oocyst loads in heterozygous transgenic Cp and Vg mosquitoes.** Cp (**A**) and Vg (**B**) heterozygous mosquitoes were fed on human blood with a standard 0.3% gametocytemia and the midguts were dissected at 8 dpi for oocysts counts. Each assay was performed with at least two biological replicates, and the oocyst loads from equal number of samples (midguts) from the different replicates were pooled for the dot-plot. Each circle represents the number of oocysts in an individual gut, and the horizontal lines (red or green) indicate the medians. *P*-values were calculated using a Mann-Whitney test by comparing to wild-type (WT) control mosquitoes.
(TIF)

Figure S3 ***P. falciparum*** **sporozoite loads in the salivary glands of wild type and hybrid transgenic mosquitoes that were provided a second blood meal.** *P. falciparum* sporozoite loads in the salivary glands (SG) of the wild type (WT) and hybrid (Hyb) transgenic mosquito lines which had either been provided a single blood meal (Non-re-fed) or provided a second naïve blood meal at 8 days after the initial *Pf*-infected blood meal (Re-fed). At least two biological replicates were included in each assay and the sporozoite numbers from equal numbers of salivary glands of different replicates were pooled for the dot-plot analysis. Each circle represents the number of sporozoites in the salivary glands of an individual mosquito, the horizontal lines (red) indicate the medians and *p*-values were calculated using a Mann-Whitney test.
(TIF)

Figure S4 **Transgenic mosquito anti-** ***P. berghei*** **activity.** *P. berghei* oocyst infection intensities of the wild type (WT), Cp, Vg, and hybrid transgenic mosquito lines at 14 dpi. At least three biological replicates were included in each assay and the oocyst loads from equal number of mosquito midguts from the different replicates were pooled for the dot-plot analysis. Each circle represents the number of oocysts in an individual mosquito, and the horizontal lines (red) indicate the medians (including zeros) and *p-*values were calculated using a Mann-Whitney as indicated here and Kruskal-Wallis (KW) test (in Table S3). Detailed statistical information of N, range, medians with or without zeros, prevalence and *p*-values from either Mann-Whitney or KW tests are presented in Table S3.
(TIF)

Figure S5 **Kaplan-Meier survivorship curve comparing transgenic (Cp, Vg) and non-transgenic (WT) mosquitoes** **after systematic bacterial infection.** Cp, Vg, and WT mosquitoes were reared under identical conditions after challenge with either Gram-negative (*E. coli*: 350,000 CFU; Ec) or Gram-positive (*S. aureus*: 210,000 CFU; Sa) bacteria at 4 dpi. Three biological replicates were shown here. Kaplan-Meier survival analysis was used together with log-rank test to determine the *p*-values, and *p*<0.05 indicates significance (Figure 3B).
(TIF)

Table S1 **Primers used for generation of constructs for embryo microinjections and the verification of transgene integration.** AgCp and AgVg denote *A. gambiae* carboxypeptidase A and vitellogenin 1 promoters respectively. Veri: verification primers. Letters in **bold**, *Italic*, or underlined in the “Primer sequence” indicate the restriction sites or the special sequences which were used for the cloning. “RE sites, Notes” indicates the restriction enzyme used with the same font format as in “Primer sequence”.
(DOC)

Table S2 **Primers used for gene expression analysis, production of PCR amplicons for** ***dsRNA*** **synthesis, and qRT-PCR validation of RNAi-mediated gene silencing and the efficiencies of gene silencing.** Underlined letters indicate the T7 promoter sequence, and the same pair of the forward (RNAiF) and the reverse primers (RNAiR) was used for both *dsRNA* synthesis and qRT-PCR expression analysis of the corresponding genes. For gene silencing validation, the different veriF (verification forward primer) and RNAiR (*dsRNA* synthesis reverse primer) were used. KD% (± SE) denotes the efficiency of gene knock-down (KD) % on average with standard error. The silencing efficiency was determined at 3 d post *dsRNA* injection, before *P. falciparum* infected blood feeding, or 24 hours post blood-feeding (24 h pbf).
(DOC)

Table S3 **Statistical analyses of ooycsts, ookinetes, or sporozoites in WT, Cp, Vg, and Hyb mosquito midguts, midgut lumens, or salivary glands, respectively; and the effect of gene silencing on** ***P. falciparum*** **infection (oocyst loads) in WT, Cp, Vg, and Hyb mosquitoes.** n: total midguts, midgut lumens or salivary glands numbers; range: range of oocysts, ookinetes, or sporozoites numbers; prevalence: % of mosquitoes with at least one parasite; Chi-square test *p*-value: for determine the significance of prevalence; median (with zeros): median oocysts, ookinetes, or sporozoites from 3 biological replicates when zeros were included; median (without zeros): median from 3 replicates when zeros were excluded; % decreased oocysts #: the % decrease in the oocyst loads; % inhibition: the % decrease in the ookinete or sporozoite loads; % increased oocysts #: the % increase in the oocyst loads. The *p*-values from Kruskal-Wallis and Mann-Whitney test are presented where *: *p*<0.05 or *p*<0.01; **: *p*<0.001; ***: *p*<0.0001; ns: no significance.
(DOC)

Table S4 **Survival analysis of transgenic and wt control mosquitoes after sugar-feeding, blood-feeding, or** ***P. falciparum*** **infected blood-feeding.**
(DOC)
