# Supplementary figures and images for: Engineered Anopheles Immunity to Plasmodium Infection
Source: PLoS Pathog. 2011 Dec 22;7(12):e1002458. doi: 10.1371/journal.ppat.1002458 (PMC3245315; doi:10.1371/journal.ppat.1002458)

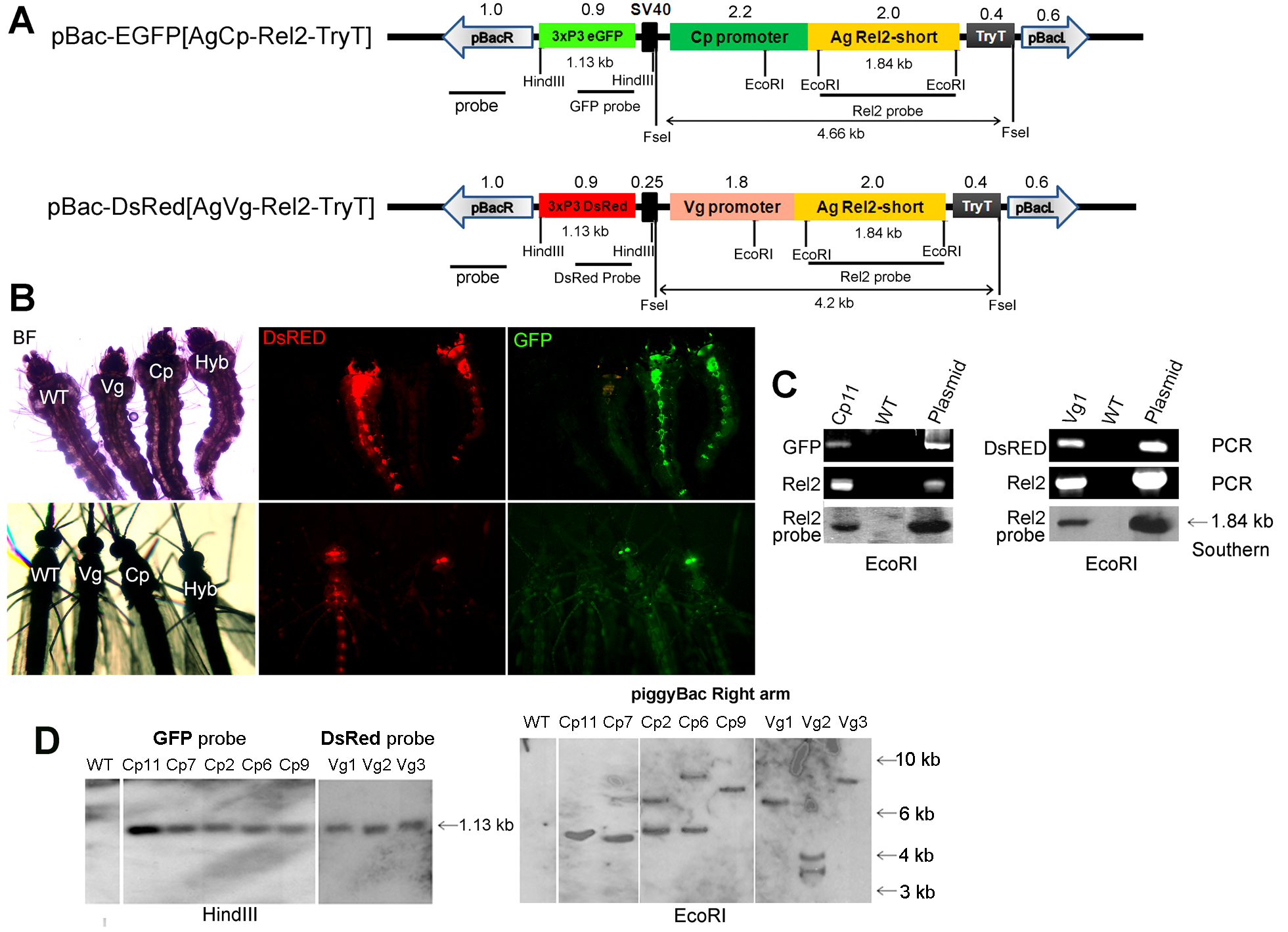

Supplement: Figure S1 — Generation of Rel2 transgenic lines in A. stephensi . (A) Schematic representation of the pBac-EGFP[AgCp-Rel2-TryT] and pBac-DsRed[AgVg-Rel2-TryT] transformation plasmids used for the germline transformation of A. stephensi. The restriction sites used for DNA digestion for Southern hybridization are indicated; the thick horizontal black lines indicate the probes (GFP, DsRed, piggybac Right arm) regions. Double-headed arrows indicate the sizes of the inserts of AgCp-Rel2 and AgVg-Rel2 for making the constructs. The sizes of each region of the corresponding constructs are indicated by the numbers above the schematic map. (B) Bright-field and fluorescent images of larvae and adults of the Cp, Vg, and Hyb transgenic lines. The control wild-type (WT) is also shown. (C) PCR confirmation and Southern blot analysis of transgene integration. In each row, column 1 shows the transgenic line DNA, column 2 the non-transgenic control WT DNA, and column 3 the plasmid DNA used in germ-line transformation. The top row is the PCR amplification of the fluorescent marker (GFP for Cp, and DsRed for Vg), and the middle row is the PCR amplification of the Rel2 transgene. The lower row is a Southern blot analysis of the Rel2 transgene on EcoRI-digested genomic DNA (gDNA) of the transgenic Cp11 and Vg1 lines, WT, and control plasmid. (D) Southern blot analysis of the genomic DNA isolated from the representative Cp and Vg transgenic strains and the wild-type control strain. HindIII and EcoRI were used for the gDNA digestion, probes from PCR products of GFP, DsRed, and piggyBac right arm were used for the Southern hybridization. WT: wild-type control parental strain; Cp: Cp lines; Vg: Vg lines. (TIF) [file ppat.1002458.s001.tif]

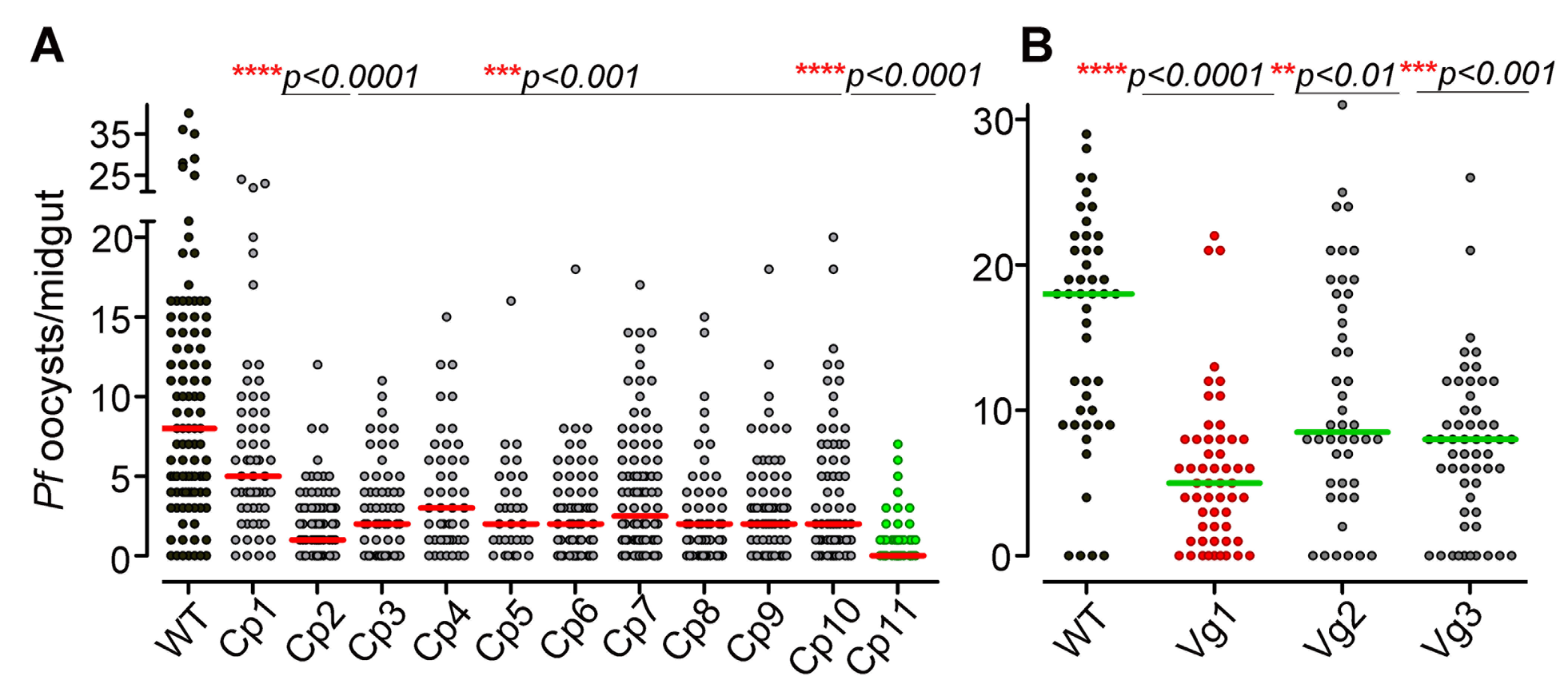

Supplement: Figure S2 — P. falciparum (NF54) oocyst loads in heterozygous transgenic Cp and Vg mosquitoes. Cp (A) and Vg (B) heterozygous mosquitoes were fed on human blood with a standard 0.3% gametocytemia and the midguts were dissected at 8 dpi for oocysts counts. Each assay was performed with at least two biological replicates, and the oocyst loads from equal number of samples (midguts) from the different replicates were pooled for the dot-plot. Each circle represents the number of oocysts in an individual gut, and the horizontal lines (red or green) indicate the medians. P-values were calculated using a Mann-Whitney test by comparing to wild-type (WT) control mosquitoes. (TIF) [file ppat.1002458.s002.tif]

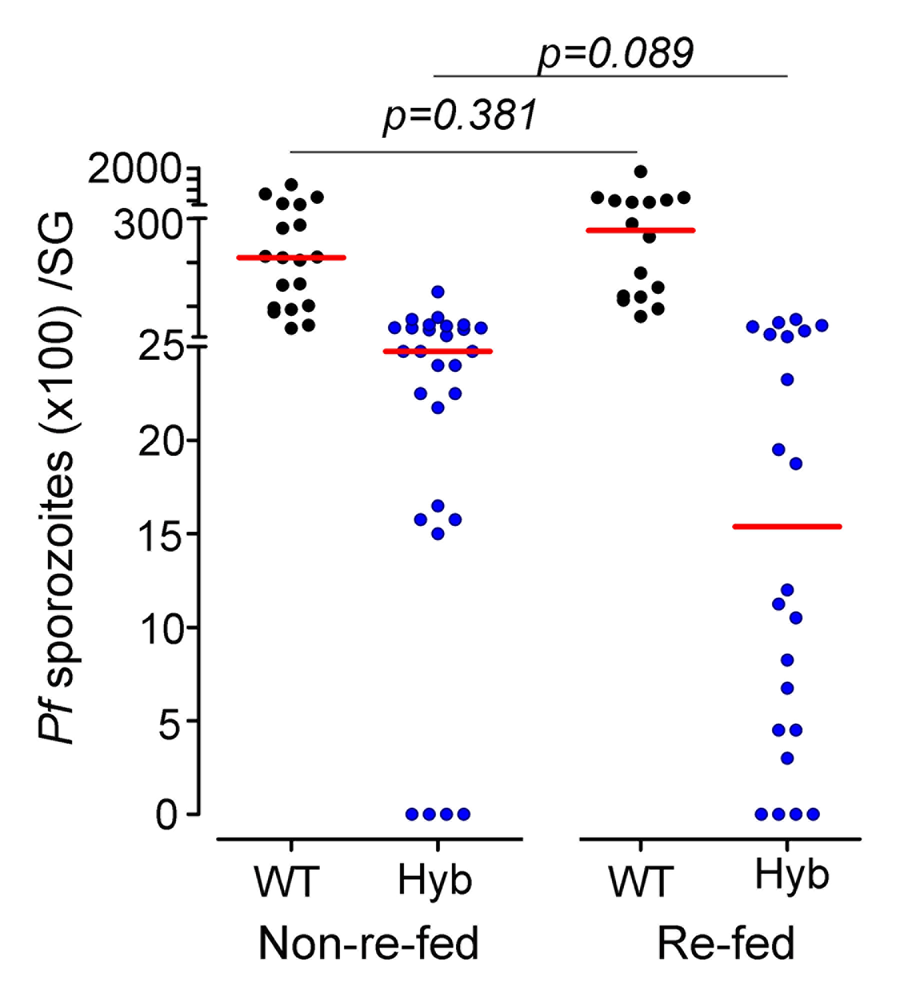

Supplement: Figure S3 — P. falciparum sporozoite loads in the salivary glands of wild type and hybrid transgenic mosquitoes that were provided a second blood meal. P. falciparum sporozoite loads in the salivary glands (SG) of the wild type (WT) and hybrid (Hyb) transgenic mosquito lines which had either been provided a single blood meal (Non-re-fed) or provided a second naïve blood meal at 8 days after the initial Pf-infected blood meal (Re-fed). At least two biological replicates were included in each assay and the sporozoite numbers from equal numbers of salivary glands of different replicates were pooled for the dot-plot analysis. Each circle represents the number of sporozoites in the salivary glands of an individual mosquito, the horizontal lines (red) indicate the medians and p-values were calculated using a Mann-Whitney test. (TIF) [file ppat.1002458.s003.tif]

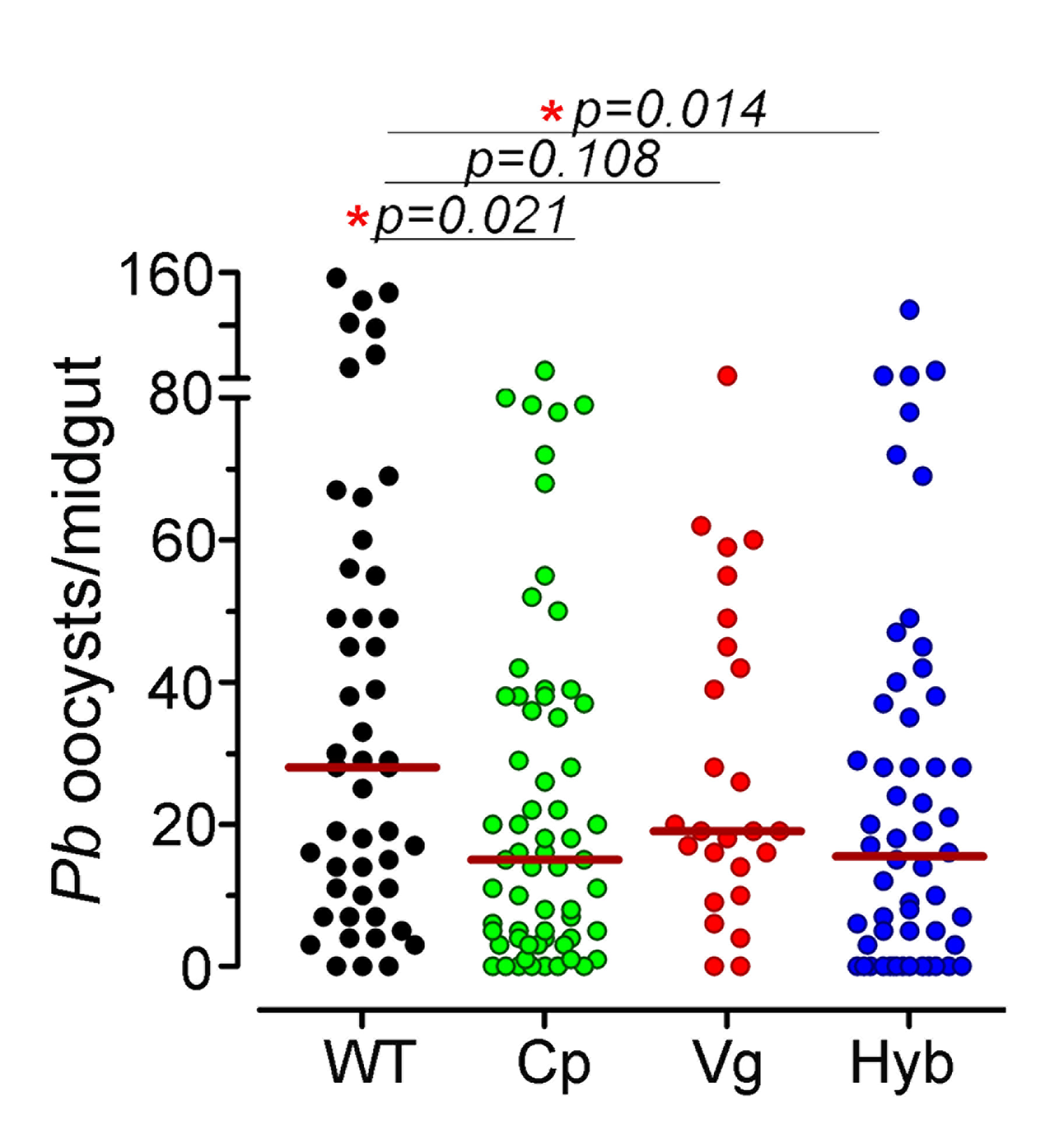

Supplement: Figure S4 — Transgenic mosquito anti- P. berghei activity. P. berghei oocyst infection intensities of the wild type (WT), Cp, Vg, and hybrid transgenic mosquito lines at 14 dpi. At least three biological replicates were included in each assay and the oocyst loads from equal number of mosquito midguts from the different replicates were pooled for the dot-plot analysis. Each circle represents the number of oocysts in an individual mosquito, and the horizontal lines (red) indicate the medians (including zeros) and p-values were calculated using a Mann-Whitney as indicated here and Kruskal-Wallis (KW) test (in Table S3). Detailed statistical information of N, range, medians with or without zeros, prevalence and p-values from either Mann-Whitney or KW tests are presented in Table S3. (TIF) [file ppat.1002458.s004.tif]

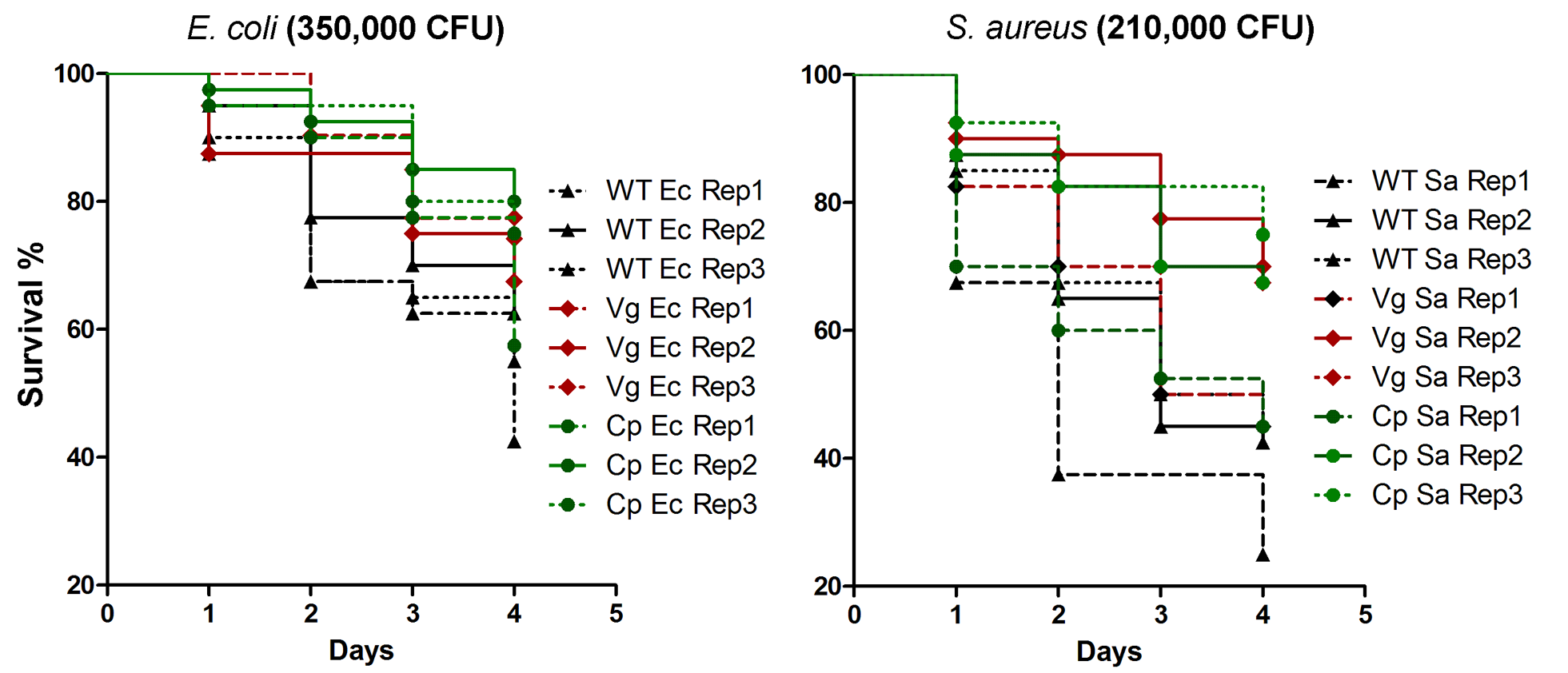

Supplement: Figure S5 — Kaplan-Meier survivorship curve comparing transgenic (Cp, Vg) and non-transgenic (WT) mosquitoes after systematic bacterial infection. Cp, Vg, and WT mosquitoes were reared under identical conditions after challenge with either Gram-negative (E. coli: 350,000 CFU; Ec) or Gram-positive (S. aureus: 210,000 CFU; Sa) bacteria at 4 dpi. Three biological replicates were shown here. Kaplan-Meier survival analysis was used together with log-rank test to determine the p-values, and p<0.05 indicates significance (Figure 3B). (TIF) [file ppat.1002458.s005.tif]
