# Supplementary material for: Engineered Anopheles Immunity to Plasmodium Infection
Source: PLoS Pathog. 2011 Dec 22;7(12):e1002458. doi: 10.1371/journal.ppat.1002458 (PMC3245315; doi:10.1371/journal.ppat.1002458)
Supplement: Table S1 — Primers used for generation of constructs for embryo microinjections and the verification of transgene integration. AgCp and AgVg denote A. gambiae carboxypeptidase A and vitellogenin 1 promoters respectively. Veri: verification primers. Letters in bold, Italic, or underlined in the “Primer sequence” indicate the restriction sites or the special sequences which were used for the cloning. “RE sites, Notes” indicates the restriction enzyme used with the same font format as in “Primer sequence”. (DOC) [file ppat.1002458.s006.doc]

Table S1. Primers used for generation of constructs for embryo microinjections and the verification of transgene integration.

| **Name** | **Primer Name** | **Primer sequence** | **RE sites, Notes** |
| --- | --- | --- | --- |
|  |  |  |  |
| **AgCp promoter** | AgCp-Pro-F | GATC***GGCCGGCC***AGGAGGCATGTCATAAGC | ***FseI*** |
|  | AgCp-Pro-R | TGGATCCGCCTCGGCCGCTTC |  |
| **AgVg promoter** | AgVg-Pro-F | TGAATGGTACC***GGCCGGCC***GACTTGTGTGCAGGACCTTTCA | KpnI, ***FseI*** |
|  | AgVg-Pro-R | AAGTAAGCTT***AGTACTGGTACGCCGTACACAACCCCA***GAGGAGGAGTAACTTCGCAATCA | HindIII, ***Vg coding seq.*** |
| **Trypsin Terminator** | Tryp-Ter-F | TGAATACTAGTTAGGTAGCTGAGCGCATGCGATCTC | SpeI |
|  | Tryp-Ter-R | TAAGTGCGGCCGC***GGCCGGCC***GGTCGGCGCGCCCACCCTTGAG | NotI, ***FseI*** |
| **Rel2-veri** | Ag Rel2-F | AGCGAGCCGGTACTGTTCAA |  |
|  | Ag Rel2-R | CATATTGTTGATTGGGTTCGA |  |
| **GFP-veri** | GFP-F | ATGGTGAGCAAGGGCGAGGAGCTGT |  |
|  | GFP-R | TTACTTGTACAGCTCGTCCATGCCG |  |
| **DsRed-veri** | DsRed-F | ACCGTGAAGCTGAAGGTGACCA |  |
|  | DsRed-R | AGGCCTCCCAGCCCATGGTCT |  |
