# Supplementary material for: Engineered Anopheles Immunity to Plasmodium Infection
Source: PLoS Pathog. 2011 Dec 22;7(12):e1002458. doi: 10.1371/journal.ppat.1002458 (PMC3245315; doi:10.1371/journal.ppat.1002458)
Supplement: Table S2 — Primers used for gene expression analysis, production of PCR amplicons for dsRNA synthesis, and qRT-PCR validation of RNAi-mediated gene silencing and the efficiencies of gene silencing. Underlined letters indicate the T7 promoter sequence, and the same pair of the forward (RNAiF) and the reverse primers (RNAiR) was used for both dsRNA synthesis and qRT-PCR expression analysis of the corresponding genes. For gene silencing validation, the different veriF (verification forward primer) and RNAiR (dsRNA synthesis reverse primer) were used. KD% (± SE) denotes the efficiency of gene knock-down (KD) % on average with standard error. The silencing efficiency was determined at 3 d post dsRNA injection, before P. falciparum infected blood feeding, or 24 hours post blood-feeding (24 h pbf). (DOC) [file ppat.1002458.s007.doc]

Table S2. Primers used for gene expression analysis, dsRNA synthesis, and qRT-PCR validation of RNAi-mediated gene silencing and the efficiencies of gene silencing.

| **Gene Name** | **Primer Name** | **Primer sequence** | **KD% (± SE)*** |
| --- | --- | --- | --- |
| **AsS7** | AsS7-F | TCGGTTCCAAGGTGATCAAAGC |  |
|  | AsS7-R | AGCGCGGTCTCTTCTGCTTGT |  |
| **AsCec3** | AsCec3-F | ACAGCCGGTAGATGGTGCGCC |  |
|  | AsCec3-R | ACGGCCTATGTTTCGTGAACC |  |
| **Gambicin** | AgGambicinT7-F | TAATACGACTCACTATAGGGCTATCTCAACCGGAAGG |  |
|  | AgGambicinT7-R | TAATACGACTCACTATAGGCCAACGTCTGGCACTGATT |  |
| **AsDefensin** | AsDefensinF | AGTCGTGGTCCTGGCGGCTCT |  |
|  | AsDefensinR | ACGAGCGATGCAATGCGCGGCA |  |
| **GFP** | GFP-RNAiF | TAATACGACTCACTATAGGATGGTGAGCAAGGGCGAGGAGCTGT |  |
|  | GFP-RNAiR | TAATACGACTCACTATAGGTTACTTGTACAGCTCGTCCATGCCG |  |
| **AsPGRP-LC** | AsPGRPLC-T7F | TAATACGACTCACTATAGGACCGTACAGGCTGTAGTTGGA | 65 (±9) ^a^  24h pbf:  72 (±8) (WT)  73 (±2) (Cp)  68 (±3) (Vg)  71 (±5) (hyb) |
|  | AsPGRPLC-T7R | TAATACGACTCACTATAGGACTCGAGGAACTTTTCCGACAT |  |
|  | AsPGRPLC-VeriF | ATGATATCGAACAGCGCTTTG |  |
| **AsTep1** | AsTep1-T7F | TAATACGACTCACTATAGGTCAGATGCGCTATCGCCAGTC | 76 (±11)^a^  24h pbf:  78 (±7) (WT)  86 (±4) (Cp)  89 (±2) (Vg)  79 (±1) (Hyb) |
|  | AsTep1-T7R | TAATACGACTCACTATAGGGCTCAGATAGGCCATTGCATT |  |
|  | AsTEP1-VeriF | CAGAATATGGTTAAGTTCGTAC |  |
| **AsLRRD7** | AsLRRD7-T7F | TAATACGACTCACTATAGGAAGCTGATCACACTCGATCTGT | 73(±14)^a^  24h pbf:  83 (±4) (WT)  88 (±7) (Cp)  89 (±2) (Vg)  81 (±2) (hyb) |
|  | AsLRRD7-T7R | TAATACGACTCACTATAGGTACGCACCATCACCGGGAACGA |  |
|  | AsLRRD7-VeriF | TCGAACCTGCGTCAGCTAGTT |  |
| **AsAPL1** | AsAPL1-T7F | TAATACGACTCACTATAGGCGTATCGAGGACGAAACGTTCC | 69(±8)^a^ |
|  | AsAPL1-T7R | TAATACGACTCACTATAGGTGATACGTACAGTCGCTCCAGA |  |
|  | AsAPL1-VeriF | TGCTTGTGCTGGACCGGAACA |  |

* KD% (± SE): gene silencing efficiency with standard error. a: gene silencing efficiency determined at 3d post *dsRNA* injection, before *P. falciparum* infected blood feeding.
