# Supplementary material for: Engineered Anopheles Immunity to Plasmodium Infection
Source: PLoS Pathog. 2011 Dec 22;7(12):e1002458. doi: 10.1371/journal.ppat.1002458 (PMC3245315; doi:10.1371/journal.ppat.1002458)
Supplement: Table S3 — Statistical analyses of ooycsts, ookinetes, or sporozoites in WT, Cp, Vg, and Hyb mosquito midguts, midgut lumens, or salivary glands, respectively; and the effect of gene silencing on P. falciparum infection (oocyst loads) in WT, Cp, Vg, and Hyb mosquitoes. n: total midguts, midgut lumens or salivary glands numbers; range: range of oocysts, ookinetes, or sporozoites numbers; prevalence: % of mosquitoes with at least one parasite; Chi-square test p-value: for determine the significance of prevalence; median (with zeros): median oocysts, ookinetes, or sporozoites from 3 biological replicates when zeros were included; median (without zeros): median from 3 replicates when zeros were excluded; % decreased oocysts #: the % decrease in the oocyst loads; % inhibition: the % decrease in the ookinete or sporozoite loads; % increased oocysts #: the % increase in the oocyst loads. The p-values from Kruskal-Wallis and Mann-Whitney test are presented where *: p<0.05 or p<0.01; **: p<0.001; ***: p<0.0001; ns: no significance. (DOC) [file ppat.1002458.s008.doc]

**Table S3. Statistical analyses of ooycst, ookinete, or sporozoite loads in WT, Cp, Vg, and Hyb mosquito** midguts, midgut lumens, or salivary glands, respectively.

| ***Fig. 1D (Pf oocysts)*** | **WT** | **Cp** | **Vg** | **Hyb** |  |
| --- | --- | --- | --- | --- | --- |
| n= | 39 | 39 | 33 | 63 |  |
| range | 0, 2-48 | 0, 1-20 | 0, 1-28 | 0, 1-20 |  |
| prevalance | 97.4% | 84.6% | 93.9% | 79.4% |  |
| Chi-square test *p-*value |  | 0.0030 | 0.4977 | 0.0001 |  |
| median (with zeros) | 17.5 | 4.0 | 9.0 | 3.0 |  |
| % decreased oocysts# |  | 77.1% | 48.6% | 82.9% |  |
| Kruskal-Wallis *p-*value | <0.0001 (***) | | | |  |
| Dunn's Multiple Comparison Summary | | *** | * | *** |  |
| Mann-Whitney test *p-*value | | <0.0001 | 0.0003 | <0.0001 |  |
| median (without zeros) | 18.0 | 4.5 | 9.0 | 4.0 |  |
| Kruskal-Wallis *p-*value | <0.0001 (***) | | | |  |
| Dunn's Multiple Comparison Summary | | *** | * | *** |  |
| Mann-Whitney test *p-*value | | <0.0001 | 0.0004 | <0.0001 |  |
| ***Fig. 1E (Pf oocysts)*** | **WT** | **Cp** | **Vg** | **Hyb** |  |
| n= | 25 | 25 | 25 | 25 |  |
| range | 0, 1-10 | 0, 1-5 | 0, 1-7 | 0, 1-6 |  |
| prevalence | 80% | 36% | 44% | 28% |  |
| Chi-square test *p-*value |  | <0.0001 | <0.0001 | <0.0001 |  |
| median (with zeros) | 3.0 | 0.0 | 0.0 | 0.0 |  |
| mean | 3.56 | 0.84 | 1.48 | 0.64 |  |
| % inhibition ( to mean) |  | 76.40% | 58.40% | 82.00% |  |
| Kruskal-Wallis *p-*value | 0.0002 (***) | | | |  |
| Dunn's Multiple Comparison Summary | | ** | * | *** |  |
| Mann-Whitney test *p-*value | | 0.0005 | 0.0082 | 0.0001 |  |
| median (without zeros) | 3.5 | 2.0 | 2.0 | 2.0 |  |
| Kruskal-Wallis *p-*value | 0.2215 (ns) | | | |  |
| Dunn's Multiple Comparison Summary | | ns | ns | ns |  |
| Mann-Whitney test *p-*value | | 0.0945 | 0.4154 | 0.1376 |  |
| ***Fig. 1F (Pf ookinetes)*** | **WT** | **Cp** | **Vg** | **Hyb** |  |
| n= | 16 | 16 | 16 | 16 |  |
| range | 168-344 | 30-250 | 90-370 | 18-216 |  |
| median (without zeros) | 228 | 144 | 200 | 118 |  |
| % inhibition |  | 36.8% | 12.3% | 48.2% |  |
| Kruskal-Wallis *p-*value | <0.0001 (***) | | | |  |
| Dunn's Multiple Comparison Summary | | ** | ns | *** |  |
| Mann-Whitney test *p-*value | | 0.0002 | 0.1807 | <0.0001 |  |
| ***Fig. 1G (Pf sporozoites)*** | **WT** | **Cp** | **Vg** | **Hyb** |  |
| n= | 23 | 13 | 16 | 23 |  |
| range | 0-, 1650-38925 | 0, 720-11525 | 0, 780-10125 | 0, 150-8475 |  |
| prevalence | 95.7% | 69.2% | 87.5% | 78.3% |  |
| Chi-squre *p-*value |  | <0.0001 | 0.0399 | 0.0002 |  |
| median (with zeros) | 6225 | 1575 | 4163 | 1650 |  |
| % inhibition |  | 74.7% | 33.1% | 73.5% |  |
| Kruskal-Wallis *p-*value | 0.0002 (***) | | | |  |
| Dunn's Multiple Comparison Summary | | ** | ns | *** |  |
| Mann-Whitney *p-*value |  | 0.0028 | 0.0384 | <0.0001 |  |
| median (without zeros) | 6300 | 2025 | 4763 | 1988 |  |
| Kruskal-Wallis *p-*value | 0.0009 (***) | | | |  |
| Dunn's Multiple Comparison Summary | | ns | ns | *** |  |
| Mann-Whitney *p-*value |  | 0.0264 | 0.062 | 0.0001 |  |
| ***fig. S4 (Pb oocysts)*** | **WT** | **Cp** | **Vg** | **Hyb** |  |
| n= | 48 | 63 | 27 | 57 |  |
| range | 0, 3-156 | 0, 1-86 | 0, 4-82 | 0, 3-132 |  |
| prevalence | 93.8% | 88.9% | 92.6% | 75.4% |  |
| Chi-square *p-*value |  | 0.3106 | 1.0000 | 0.0003 |  |
| median (with zeros) | 28.0 | 15.0 | 19.0 | 15.5 |  |
| % inhibition |  | 46.4% | 32.1% | 44.6% |  |
| Kruskal-Wallis *p-*value | 0.0363 (*) | | | |  |
| Dunn's Multiple Comparison Summary |  | ns | ns | ns |  |
| Mann-Whitney *p-*value |  | 0.0209 | 0.1082 | 0.0142 |  |
| median (without zeros) | 29.0 | 18.0 | 19.5 | 23.5 |  |
| Kruskal-Wallis *p-*value | 0.1461 (ns) | | | |  |
| Dunn's Multiple Comparison Summary | | ns | ns | ns |  |
| Mann-Whitney *p-*value |  | 0.0332 | 0.5775 | 0.3079 |  |
|  |  |  |  |  |  |
| ***Fig. 2*** | ***dsGFP*** | ***dsTEP1*** | ***dsAPL1*** | ***dsLRRD7*** | ***dsPGRP-LC*** |
| **WT** |  |  |  |  |  |
| n= | 90 | 54 | 45 | 48 | 63 |
| range | 0, 7-107 | 0, 7-103 | 0, 20-102 | 0, 6-111 | 0, 1-80 |
| prevalence | 85.6% | 85.2% | 91.1% | 91.7% | 87.3% |
| median (with zeros) | 19.0 | 35.0 | 42.0 | 36.0 | 30.0 |
| % increased oocysts load |  | 84.2% | 121.1% | 89.5% | 57.9% |
| Kruskal-Wallis *p-*value | 0.0012 (**) | | | | |
| Dunn's Multiple Comparison Summary | | * | ** | * | ns |
| Mann-Whitney *p-*value |  | 0.0078 | 0.0001 | 0.005 | 0.0743 |
| **Cp** |  |  |  |  |  |
| n= | 78 | 48 | 66 | 51 | 48 |
| range | 0, 1-48 | 0, 4-90 | 0, 4-69 | 0, 1-85 | 0, 1-69 |
| prevalence | 75.6% | 83.3% | 97.0% | 90.2% | 72.9% |
| median | 6 | 16 | 18 | 14.5 | 11 |
| % increased oocysts load |  | 166.7% | 200.0% | 141.7% | 83.3% |
| Kruskal-Wallis *p-*value | <0.0001 (***) | | | | |
| Dunn's Multiple Comparison Summary | | * | *** | ** | ns |
| Mann-Whitney *p-*value |  | 0.0028 | <0.0001 | 0.0012 | 0.5645 |
| **Vg** |  |  |  |  |  |
| n= | 60 | 48 | 66 | 48 | 78 |
| range | 0, 2-58 | 0, 2-74 | 5-62 | 0, 2-82 | 0, 2-72 |
| prevalence | 78.3% | 91.7% | 100.0% | 97.9% | 84.6% |
| median | 13.0 | 18.0 | 31.0 | 29.0 | 14.0 |
| % increased oocysts load |  | 38.5% | 138.5% | 123.1% | 7.7% |
| Kruskal-Wallis *p-*value | <0.0001 (***) | | | | |
| Dunn's Multiple Comparison Summary | | ns | *** | *** | ns |
| Mann-Whitney *p-*value |  | 0.0304 | <0.0001 | <0.0001 | 0.2822 |
| **Hyb** |  |  |  |  |  |
| n= | 72 | 90 | 57 | 42 | 51 |
| range | 0, 2-34 | 0, 1-77 | 0, 4-59 | 0, 3-43 | 0, 1-28 |
| prevalence | 76.4% | 95.6% | 93.0% | 83.3% | 90.2% |
| median | 6.0 | 13.0 | 13.0 | 12.0 | 9.0 |
| % increased oocysts load |  | 116.7% | 116.7% | 100.0% | 50.0% |
| Kruskal-Wallis *p-*value | <0.0001 (***) | | | | |
| Dunn's Multiple Comparison Summary | | ** | *** | ns | ns |
| Mann-Whitney *p-*value |  | 0.0009 | <0.0001 | 0.0111 | 0.3096 |
|  |  |  |  |  |  |
| ***Fig. 3C*** | **Septic** | | **Aseptic** | |  |
|  | WT | Hyb | WT | Hyb |  |
| n= | 87 | 63 | 90 | 60 |  |
| range | 0, 2-92 | 0, 1-38 | 0, 1-148 | 0, 1-37 |  |
| prevalence | 97.7% | 88.9% | 98.9% | 88.3% |  |
| median | 15 | 7 | 29 | 8 |  |
| % inhibition |  | 53.3% |  | 72.4% |  |
| Mann-Whitney *p-*value |  | <0.0001 |  | <0.0001 |  |
|  |  |  |  |  |  |
| ***Fig. 3E*** | **WT** | **Cp** | **Vg** | **Hyb** |  |
| n= | 70 | 70 | 70 | 70 |  |
| range | 0-162 | 0-129 | 0-129 | 0-167 |  |
| median | 56 | 42 | 38 | 45 |  |
| Mann-Whitney *p-*value |  | 0.1294 | 0.0115 | 0.1463 |  |
