# Supplementary material for: Engineered Anopheles Immunity to Plasmodium Infection
Source: PLoS Pathog. 2011 Dec 22;7(12):e1002458. doi: 10.1371/journal.ppat.1002458 (PMC3245315; doi:10.1371/journal.ppat.1002458)
Supplement: Table S4 — Survival analysis of transgenic and wt control mosquitoes after sugar-feeding, blood-feeding, or P. falciparum infected blood-feeding. (DOC) [file ppat.1002458.s009.doc]

**Table S4. Survival analysis of transgenic and wt control mosquitoes after sugar-feeding, blood-feeding, or *P. falciparum*** infected blood-feeding.

| **Longevity of transgenic mosquitoes (10% sucrose-fed)** | | | | | | | | | | | |
| --- | --- | --- | --- | --- | --- | --- | --- | --- | --- | --- | --- |
|  | **wt control** | | **Cp-Rel2** | | **Wilcoxon** | **Vg-Rel2** | | **Wilcoxon** | **Hyb-Rel2** | | **Wilcoxon** |
| Exp. | N | Median | N | Median | *p** | N | Median | *p* | N | Median | *p* |
| #1 | 50 | 20.0 | 50 | 22.5 | 0.0136 | 50 | 21.0 | 0.0733 | 50 | 20.0 | 0.0783 |
| #2 | 50 | 18.0 | 50 | 20.0 | 0.0926 | 50 | 20.0 | 0.7751 | 50 | 20.0 | 0.1626 |
| #3 | 50 | 18.0 | 50 | 21.0 | 0.1013 | 50 | 19.0 | 0.3461 | 50 | 19.0 | 0.2536 |
| **Longevity of transgenic mosquitoes (a single naïve blood meal fed, then 10% sucrose)** | | | | | | | | | | | |
|  | **wt control** | | **Cp-Rel2** | | **Wilcoxon** | **Vg-Rel2** | | **Wilcoxon** | **Hyb-Rel2** | | **Wilcoxon** |
| Exp. | N | Median | N | Median | *p** | N | Median | *p* | N | Median | *p** |
| #1 | 60 | 16.0 | 55 | 22.0 | <0.0001 | 50 | 16.0 | 0.8037 | 65 | 17.0 | 0.0357 |
| #2 | 60 | 16.0 | 55 | 16.0 | 0.4085 | 50 | 13.0 | 0.0536 | 65 | 15.0 | 0.2174 |
| #3 | 60 | 16.5 | 55 | 16.0 | 0.5849 | 50 | 16.0 | 0.6065 | 65 | 19.0 | 0.0371 |
| **Longevity of transgenic mosquitoes (multiple naïve blood meals, then 10% sucrose)** | | | | | | | | | | | |
|  | **wt control** | | **Cp-Rel2** | | **Wilcoxon** | **Vg-Rel2** | | **Wilcoxon** | **Hyb-Rel2** | | **Wilcoxon** |
| Exp. | N | Median | N | Median | *p** | N | Median | *p* | N | Median | *p* |
| #1 | 74 | 17.0 | 77 | 20.0 | 0.0757 | 68 | 16.0 | 0.7538 | 68 | 15.0 | 0.0992 |
| #2 | 74 | 17.0 | 68 | 19.0 | 0.7711 | 68 | 18.0 | 0.9861 | 68 | 16.0 | 0.3562 |
| #3 | 74 | 17.0 | 65 | 21.0 | 0.0003 | 68 | 16.0 | 0.5197 | 68 | 17.0 | 0.4744 |
| **Longevity of transgenic mosquitoes (*P. falciparum*-infected blood-fed, then 10% sucrose)** | | | | | | | | | | | |
|  | **wt control** | | **Cp-Rel2** | | **Wilcoxon** | **Vg-Rel2** | | **Wilcoxon** | **Hyb-Rel2** | | **Wilcoxon** |
| Exp. | N | Median | N | Median | *p** | N | Median | *p** | N | Median | *p** |
| #1 | 55 | 18.0 | 55 | 16.0 | 0.0453 | 58 | 14.0 | 0.0067 | 60 | 15.0 | 0.0302 |
| #2 | 55 | 17.0 | 55 | 17.0 | 0.9794 | 58 | 14.0 | 0.8155 | 60 | 14.0 | 0.0298 |
| #3 | 55 | 18.0 | 55 | 16.0 | 0.2963 | 58 | 15.0 | 0.0711 | 60 | 13.0 | <0.0001 |

p*: the blue highlighted wells indicate the significance of survival rates between the wt control and transgenic mosquitoes.
